# Place cells dynamically refine grid cell activities to reduce error accumulation during path integration in a continuous attractor model

**DOI:** 10.1038/s41598-022-25863-2

**Published:** 2022-12-12

**Authors:** Jose A. Fernandez-Leon, Ahmet Kerim Uysal, Daoyun Ji

**Affiliations:** 1 Department of Neuroscience, Baylor College of Medicine, Houston, TX USA; 2 Universidad Nacional del Centro de la Provincia de Buenos Aires (UNCPBA), Facultad de Ciencias Exactas, INTIA, Tandil, Buenos Aires Argentina; 3 CIFICEN, UNCPBA-CICPBA-CONICET, Tandil, Argentina; 4 Consejo Nacional de Investigaciones Científicas y Técnicas (CONICET), Buenos Aires, Argentina

**Keywords:** Cognitive neuroscience, Computational neuroscience, Network models, Spatial memory

## Abstract

Navigation is one of the most fundamental skills of animals. During spatial navigation, grid cells in the medial entorhinal cortex process speed and direction of the animal to map the environment. Hippocampal place cells, in turn, encode place using sensory signals and reduce the accumulated error of grid cells for path integration. Although both cell types are part of the path integration system, the dynamic relationship between place and grid cells and the error reduction mechanism is yet to be understood. We implemented a realistic model of grid cells based on a continuous attractor model. The grid cell model was coupled to a place cell model to address their dynamic relationship during a simulated animal’s exploration of a square arena. The grid cell model processed the animal’s velocity and place field information from place cells. Place cells incorporated salient visual features and proximity information with input from grid cells to define their place fields. Grid cells had similar spatial phases but a diversity of spacings and orientations. To determine the role of place cells in error reduction for path integration, the animal’s position estimates were decoded from grid cell activities with and without the place field input. We found that the accumulated error was reduced as place fields emerged during the exploration. Place fields closer to the animal’s current location contributed more to the error reduction than remote place fields. Place cells’ fields encoding space could function as spatial anchoring signals for precise path integration by grid cells.

## Introduction

To navigate successfully, an animal needs to create an internal “cognitive map,” a mental representation of the outside world^1^. This mental representation is known to be sustained by several brain regions and various cell types, building the internal navigation system.

Some of the main components of the internal navigation system are the place cells in the hippocampus and grid cells in the medial entorhinal cortex (MEC)^2^. The hippocampal place cells code where the animal is in space^3,4^ by tuning their firing activity to a specific location in the environment, creating a place field^5^. Such tuning requires sensory inputs, i.e. from the visual cortex^6,7^, and inputs from sensory-independent spatial metrics, including the MEC^5,8^.

The sensory-independent spatial metric of MEC is coded by grid cells that make a “coordinate map” in the brain^9^. Grid cells display a striking hexagonal firing pattern in an open field, creating multiple firing fields (i.e., places where the grid cells fire at regular intervals as an animal navigates an open area)^2^. Grid cells’ activity is modulated by the speed and direction of running, suggesting that they combine idiothetic (self-motion) cues to signal distance and direction information necessary for path integration^2,10–13^.

Path integration is a navigational strategy that animals use to estimate their distance and orientation relative to a starting location based on idiothetic cues^14^. Path integration usually conveys an inaccurate estimation of the animal's location due to rapid error accumulation in grid cells. With ongoing exploration, this accumulation of error in terms of the distance between the estimated location and the animal’s current position increases^15,16^.

For a successful navigation, error accumulation during path integration must be decreased^17,18^. It was predicted that environmental landmarks could anchor grid cells, helping the error accumulation get reduced^19^. Furthermore, Hardcastle et al.^18^ showed that environmental boundaries act as one type of landmark to reduce the error accumulation in grid cells. Theoretically, Mulas et al.^20^ proposed an error reduction explanation, based on Hebbian plasticity between sensory inputs and the grid cell network. Hebbian plasticity in their model realigned the grid cells’ drifted activity back to the agent’s correct location and reduced the accumulated error.

Theoretical studies usually assumed that error reduction occurs intrinsically in grid cell networks without a possible role of place cells, despite anatomic evidence of connectivity between the hippocampus and MEC^21–26^. However, the inactivation of one region affects the dynamics of the other, such as an extinguished grid pattern after hippocampus silencing^27^, or a decreased place field stability after MEC inactivation^28^. Regarding brain connectivity, studies also reported that a significant portion of excitatory afferent projections of MEC originates in grid cells and projects to place cells^29–31^. Large feedback projections from place cells to the deep layers of MEC were similarly reported^32^.

The functional organization between these two brain regions was long studied, discussing whether the hippocampus or MEC drives the activity of the other region^33–37^. Several works arose on how grid cells and place cells could affect each other^38–52^. Recently, theoretical studies have started characterizing the dynamic relationship between the hippocampus and MEC with a network of loops^24,53^ and a reciprocal connectivity that enables the error reduction mechanism for path integration^54^.

Given the experimental and theoretical evidence presented, we hypothesize that the key to error reduction is the dynamic coupling between place and grid cells, which would allow the integration of idiothetic (internal) and allothetic (external) cues to calibrate spatial representations for path integration. We tested our hypothesis through a computational simulation by combining place cell and grid cell models that dynamically inform each other. The place cell model uniquely integrated two aspects, one mimicking the emergence of place fields from an idiothetic energy viewpoint and distal (proximity) information^55^, and another mimicking allocentric place field emergence based on vision^56^. The grid cell model represented different grid modules resembling the topographical organization in the dorsocaudal MEC^57,58^, which considered the grid's orientation and phase through the spacing (minimal inter-subfields distance) and the size of its subfields^17^. Grid modules along the dorso-ventral axis of the superficial layers of MEC have different spatial scales^59,60^. However, it is also known from the literature^59^ that grid cells within each grid cell module share the same spatial scale. We focused on examining whether the simulated grid cells in different modules benefited from the simulated place cells differently to reduce errors, in which the effects of having diverse scales could reveal a function of a scaling property on locating the animal in space. Coupling the grid and place cell models, we studied the grid cells’ error reduction dynamics for path integration embedded into the grid cell network.

## Results

### Salient visual features enabled the place cell’s fields to emerge

**Figure 1:**
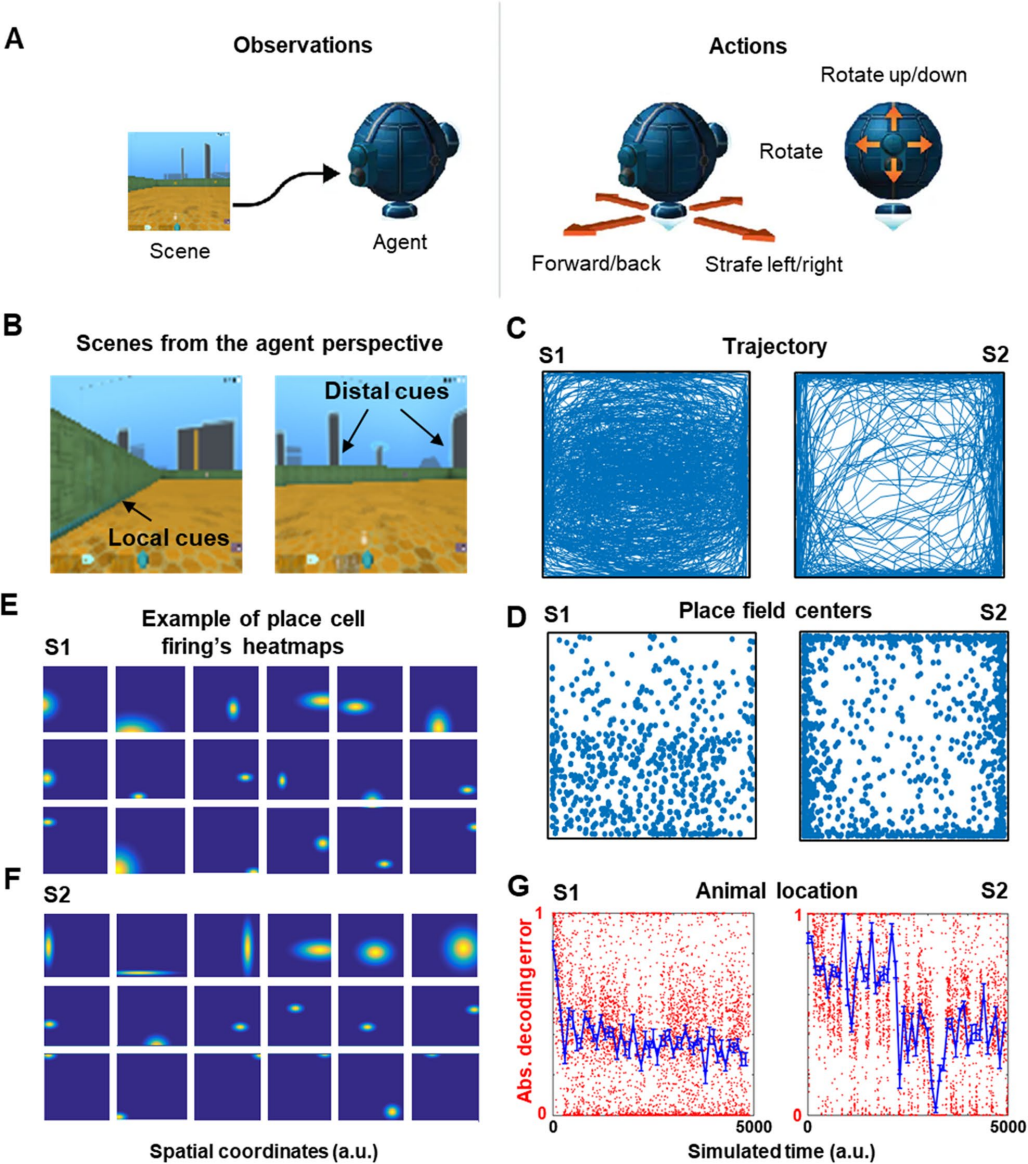
Place field formation from visual scenes enabled estimating the animal’s position. (**A**) Schematic of the simulated animal. (**B**) Examples of the animal’s perspective images showing local and distal cues. (**A**) and (**B**) were adapted from Google DeepMind Lab open source software (https://www.deepmind.com/open-source/deepmind-lab)^61^. (**C**) Two typical example trajectories (S1 and S2) during random exploration of a 2D square arena. (**D**) Place field centers for S1 and S2. (**E**,**F**) Example place fields for S1 and S2 at different locations with different field sizes. (**G**) Example of absolute decoding error from the place cell population along the navigation. Plots indicated that there was a tendency for location error to decrease along the simulation for both trajectories. Blue: average decoding error in 100-time bins across the simulated time; error bars represent s.e.m. All panels except for (**A**) and (**B**) were made using custom code in Matlab R2016b (https://www.mathworks.com/). This work is licensed under a Creative Commons Attribution 4.0 (CC BY 4.0) International License (https://creativecommons.org/licenses/by/4.0/).

We have employed DeepMind Lab^61^ to simulate the spatial exploration of a rodent-like organism in a square arena (animal in brief from now on) (Fig. 1A). We took visual scenes from the animal’s perspective during random exploration (Fig. 1B) to identify salient visual features that elicited activity in place cells. The DeepMind Lab simulator allowed the animal to move and adapt to a configurable synthetic environment. Across all the simulations presented here, the animal’s navigation was only controlled by the search-and-seek factory definition provided in the simulator^61^, but not by the place or grid cell networks.

We registered the movements across the spatial exploration (from a zenithal standpoint) of the square arena without goals (i.e., food or water for a rat). Two example trajectories of random exploration with different strategies are shown in Fig. 1C: a trajectory displaying mostly unevenly distributed paths around the center of the arena (S1), and a trajectory primarily close to the walls (S2). In both cases, the animal covered the entire arena over time. There was no information carried over from one exploration to another because all the synaptic weights of both networks were erased between experiments. The rationality of testing our hypothesis based on S1 and S2 was that these trajectories are typically observed in rodents in an open field. Based on S1 and S2, we quantified place and grid cell properties and path integration.

A fully connected feed-forward network was implemented to determine activations of place cells and sensors as presented in^62^. Visual scenes were used to train the place cell network post navigation, speeding up the learning process of the place cell network and statistical analyses. Based on Hodgkin-Huxley (H–H) model with metabolic requirements and energy consumption features (Suppl. Fig. 1), the place cell network linked proximity sensors (${N}_{s}=3$) to place cells (${N}_{pc}$ = 2000) through synaptic-like weights (Suppl. Fig. 2A,E). Sensors measured spatial distances (proximity) between the animal’s current position and walls. A measured distance value was sent from each proximity sensor to every place cell through different synaptic weights. These synaptic weights coded the relationship between proximity and place cell activation which affected the firing of place cells. Every place cell responding to the current location with a firing power above a certain threshold modified their weights from sensors through a learning rule as reported in^63^. Weights were the basis vectors in the place cell model that  computed the firing powers of these neurons^55,62^. Using the firing rate definition as in^64^ and ^65^, we considered the energy consumption of a place cell during an action potential (or spike)^62^. Place cells also detected whether preferred visual features appeared on scenes to maximize place field emergence when detecting these features from the sensed environment^56^.

For a place field to emerge on the animal’s current position, three conditions must be met simultaneously: (i) a place cell must fire as a product of the dynamics of the place cell network based on the H–H model (where each neuron receives proximity information)^55,62^; (ii) a place cell must receive enough input from grid modules (see^39^ for physiological details); (iii) preferred features of the place cell must match features detected in the visual scene^56^. Only when these conditions are met simultaneously, will a place field on the animal’s current position emerge (see Suppl. Fig. 8). More details are provided as follows.

Place cells had individual differences from each other, such as sensory preferences for detecting salient features and metabolic-like features (Suppl. Fig. 2B,F). These differences caused the firing power (Suppl. Fig. 2C,G) and the distribution of maximum firing power across neurons (Suppl. Fig. 2D,H) vary along the network during the simulation. The place fields were defined as the set of all the locations with firing power larger than a predefined value. Each place cell acquired a unique spatial selectivity to form its place field from the detected salient visual features, where most cells developed fewer place fields (Suppl. Fig. 2B,F). This selectivity occurred according to the neurons’ randomly defined activation preferences. Furthermore, some neurons showed sparse firing (i.e., firing rates with a highly peaked, non-Gaussian distribution) that defined their place fields, while other neurons presented firing profiles across the arena or close to corners for both trajectories (S1: Fig. 1E; S2: Fig. 1F). We observed a non-uniform distribution of place field centers across the arena for S1 trajectory (Fig. 1D-left), and mostly along the walls for S2 trajectory (Fig. 1D-right).

In addition, we evaluated whether place fields conveyed precise spatial information to estimate the animal’s position under different trajectories. The estimated location error (Loc) of the animal from place cell activity was computed by the weighted average of place field centers according to the response set by where the place fields emerged, and by the activity power of neurons at each time step. The Loc was normalized using the joint activity power of all neurons^62^. The error between the actual position and the estimated position from the place cell activities was profiled using 100 bins to characterize the absolute decoding error average (S1: Fig. 1G-left; S2: Fig. 1G-right). In general, we observed that for both trajectories there was a tendency for the location error to decrease over time, but the average was more variable for the S2 trajectory. In this model, a decoding error of 1 indicates that most place neurons fired initially without selectivity to a certain location. As the place cells received different inputs regarding proximity information from sensory neurons, they started selective firing in the arena. Therefore, the normalized Loc over joint activity power of all neurons got closer to 0.

### Place fields emergence remained stable after the animal’s early exploration

Having shown that place cells can detect salient features from visual scenes as local or distal reference points, we analyzed the spatial distribution of place fields at time $t$ for both trajectories, S1 and S2, using entropy $H$ (i.e., Shannon’s entropy), spatial entropy $S$ and information density $Z$. $H$ measured information on how the arena was encoded by the population of place cells. $S$ measured how random the place fields were distributed in the arena. Since $H$ was related to the number and distribution of the place fields, $Z$ measured $H$ per place in the area (see “Methods” for details). A change in $H$, $S$, or $Z$ measured how much information was gained/lost about the arena. As the animal explored the arena more, place fields would get stabilized, and the change in information would be less. By looking at the dynamics of the change, we were able to infer the formation dynamics of the place fields, but more importantly, we were able to infer the stability of place fields.

**Figure 2:**
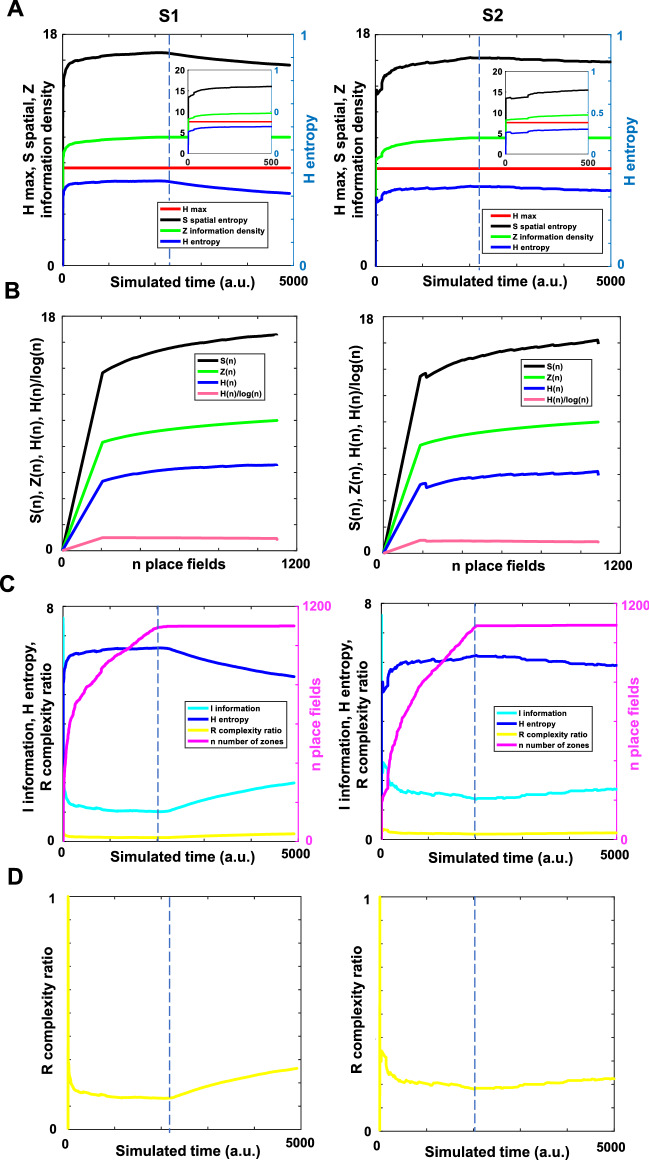
Place field emergence stabilized after the animal's early exploration. (**A**) Examples of entropy distributions across the simulated time. Insets represent the same data between 0 and 500 simulated time (a.u.). (**B**) Entropy statistics against the increase in the number of place fields during navigation. *H* is entropy, *S* is spatial entropy, and *Z* is information density. (**C**) Changes in information *I,* entropy *H*, number of place fields *n*, and complexity ratio *R* in the population of place cells across time. (**D**) Representational complexity stabilized after early exploration. The blue dashed line represents the initial time when a stable number of place fields was established. All panels were made using custom code in Matlab R2016b (https://www.mathworks.com/). This work is licensed under a Creative Commons Attribution 4.0 (CC BY 4.0) International License (https://creativecommons.org/licenses/by/4.0/).

As shown in Fig. 2A, $S$ and $Z$ sharply increased in magnitude after a few simulated time steps (i.e., ~ $t$ = 20), approaching their maximum value from ~ $t$ = 2000 on. For ~ $t$ < 2000 in Fig. 2A in both trajectories, small reversals with entropy $H$ and information density $Z$ were observed (insets in Fig. 2A, left and right). The reversal effect was largely due to the dynamics of the emerged place fields that increased in number non-uniformly overtime. For ~ $t$ > 2000 in Fig. 2A, left and right, there was a decay in $H$ and $S$, but not for $Z$. This decay suggested that no further spatial information was acquired after a certain number of place fields emerged. However, these measurements were difficult to interpret because they did not show strong links to the density distributions of place fields given the animal’s trajectories. This was also the case for Fig. 1G, sharp decreases were observed in the mean estimated location error for both trajectories. Still, for S1 the error remained relatively less variable than for the S2 trajectory across time.

Thus, we next examined changes in the place field distribution when the number of place fields ($n$) were increased during the sampling of the scenes. We plotted the $H$ entropy and its decomposition into $S$ spatial entropy and $Z$ information density against the place field number $n$ in Fig. 2B. We did not see significant differences between S1 and S2 trajectories for these measures. This relationship revealed that despite some marginal changes, $n$ is by far the largest determinant of the level of complexity (refer to^66^ for an associated measure).

Because the distribution of place fields was largely due to changes in the number of fields, we decided to analyze further the changes in place field distribution through time. As shown in Fig. 2C for both S1 and S2, the number of place fields across time increased till late exploration (~ $t$ = 2000), and then remained stable. Reversals in value $H$ were related to the reversals in the number of emerged place fields (Fig. 2C). With a small number of place fields (i.e., when the animal did not yet cover much space exploring the arena), we should assume then that the changes in entropy were proportionately larger by simply reflecting on changes in the maximum entropies $(log\left(n\right)+1-log\left(n\right))/log(n)$, which gets smaller as $n$ increases.

Figure 2C also showed that the growth in the number of place fields $n$ dominated the change in information *I* for early exploration. This change was correlated with an early decay in $I$ (i.e., ~ $t$ < 20) because adding place fields impacted the place field spatial information across timeless. There was no further change in the information $I$ after the number of place fields reached a plateau around $t$ = 2000. This time point ($t$ = 2000) represented when the place fields were stabilized after repeated exploration of the environment. Our analyses suggested that till $t$ = 2000, some place fields emerged fast, while others emerged late, which is similar to empirical findings. For example, when a rat runs a simple linear track, some place fields are formed only after several laps of run even if the rat had explored the linear track in the first run. In our simulation, although the animal had explored all parts of the arena, it took $t$ = 2000-time steps for place fields to cover the arena and have a stable representation as a population.

Comparing the $H$ entropy for $t$ < 2000 vs. t > 2000, we found that the entropy before and after reaching the stability represented different distributions for the S1 trajectory (*p* < 1e−200; Mann–Whitney U test; Fig. 2C). However, when performed the same analysis for the S2 trajectory, we observed that the $H$ entropy did not represent a clearly different distribution before and after reaching place field stability (*p* = 0.03; Mann–Whitney U test when assuming *p* < 0.01; Fig. 2C).

Computing the complement of the ratio between the entropy and its maximum value, we also defined a complexity ratio $R$, which revealed the percentage of information needed at any period to move to maximum complexity of place field distribution and plotted it across the number of emergent place fields (Fig. 2C and magnified in Fig. 2D). At the beginning of the simulation (~ $t$ < 20), the number of place fields was relatively low and then increased (till ~$t=$ 2000) as the animal explored the arena. Examining the complexity ratio $R$, it increased quickly and reached a plateau in early exploration, but afterward continued increasing. The late increase after reaching plateau happened for both trajectories steadily but only significantly for the S1 trajectory (S1: p < 1e−200; S2: p = 0.44; Mann–Whitney U test; $t$ in 100-to-2000-time steps vs. in 2001-to-end of the simulation Fig. 2D). Importantly, $R$ indicated how significant the effect of emerging place fields was on the complexity measures. This observation was also reflected in the value of $H$, which correlated with the complexity ratio as illustrated in Fig. 2C (the mean complexity ratio is illustrated in Fig. 2D).

A comparison of the place field peak location per time step was performed considering the distance of the peak between two consecutive time steps. Supp. Fig. 3 showed that the mean change in place field peak location (Δ PF peak location) was significantly higher before place fields reached stability (t = 2000; S1: p = 9.50e−135; S2: p = 4.74e−153; Two-sample t-test). These analyses complemented those reported in Fig. 2 in that stability of place fields was reached after initial exploration.

Overall, these results showed a fast growth in the number of place fields from the start of the simulation. A sufficient pool of place cells after early exploration with their individual preferences for detecting salient features in visual scenes enabled space coding.

### Grid patterns were resilient but contingent on the animal’s trajectory

**Figure 3:**
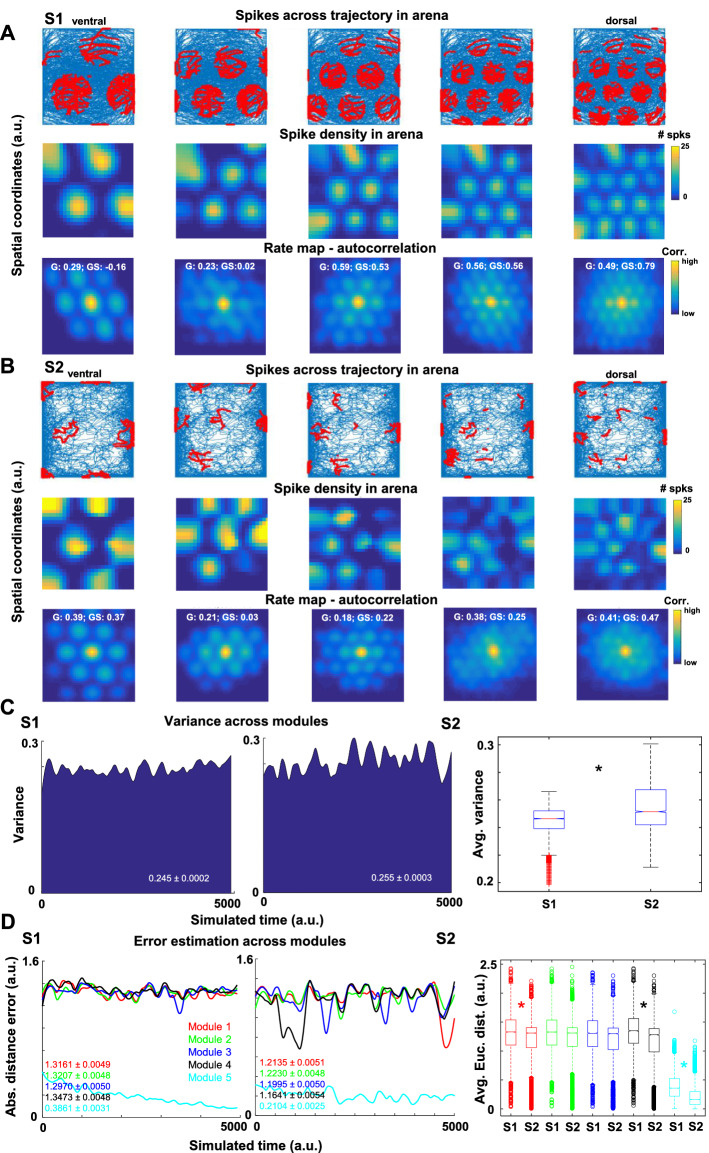
Grid patterns were maintained across the animal’s trajectory but depended on the trajectory features. (**A**,**B**) Spikes across trajectories, spike density and rate map plots from dorsal (right side) to ventral (left side) grid network configurations. Note that for each column, the plots indicate the same neuron (5 different neurons in total separately for (**A**) and (**B**) groups of plots). Regular triangular tessellation can be observed for both trajectories. Yellow represents maximum activity in the spike density and rate map plots, and red indicates spike clusters of the same neurons as shown in the spike density plots along the trajectory. Over each rate map, the gridness score (G) and the squared gridness (GS) are shown. (**C**) Variance across modules over place estimates through time for both trajectories. (**D**) The Euclidean distance between estimates for each module from dorsal (module 1) to ventral (module 5) modules. The inset numbers represent mean ± sem for the associated data for all the panels. All panels were made using custom code in Matlab R2016b (https://www.mathworks.com/). This work is licensed under a Creative Commons Attribution 4.0 (CC BY 4.0) International License (https://creativecommons.org/licenses/by/4.0/).

We next computed mean activity maps (spike densities across the arena; Fig. 3A,B-upper two rows) of grid cells as a function of the animal’s position, and the rate map autocorrelation (Fig. 3A,B-bottom row) for each dorsal to the ventral module in network organization (S1: Fig. 3A; S2: Fig. 3B). For trajectory S1, these maps showed a coherent and stable activity of the multiple grid subfields from dorsal to ventral modules with visually clear, regular triangular tessellating subfields (Fig. 3A). For trajectory S2, since the animal remained closer to walls and less often visited the middle of the arena, we expected a visually less rich grid firing pattern, meaning a less visible but still present triangular tessellation. This phenomenon was shown in the rate (autocorrelations) maps in Fig. 3B. The spatial periodicity of grid cells firing was maintained in S2 (Fig. 3B, bottom row), but grid formation was distorted because the animal did not uniformly cover the arena (Fig. 3B, top row). Importantly, our observation of the S2 trajectory can be correlated to recording sessions where animals move in 1D circular tracks^67^, as well as an animal exploring the whole arena before observing the characteristic pattern of grid cells^52^; c.f.^68^.

To compare the tessellations of grid cells between trajectories, we examined the distribution of the hexagonal grids using a mean (hexagonal) gridness score^11^. This score measured the degree to which a hexagonal grid resembled a hexagonal pattern during the simulations. Considering all the grid cells, we noticed that the mean gridness score was higher for the S1 than the S2 trajectory (S1: 0.41 ± 0.003; S2: 0.38 ± 0.003; Supp. Fig. 4). A comparison between the gridness distributions for S1 indicated that the mean was significantly different than S2 (p = 1.2e−12; Two-sample t-test), and both trajectories represented different distributions (p = 1.6e−32; Two-sample Kolmogorov–Smirnov test). Based on^69^, we also measured a “squareness gridness” score; i.e., the squareness of the grids was measured as opposed to ‘hexagonality’. The rationale for using the squareness gridness score was to evaluate if the trajectory affected the convergence to hexagons. Our motivation for using the squareness measure was that an initial visual inspection of grid rate maps (Fig. 3A,B) suggested there were deformations of the gridness across modules and trajectories. We found a higher square-gridness score for the S1 trajectory compared to the S2 trajectory (S1: 0.45 ± 0.008; S2: 0.39 ± 0.007; mean ± sem; Supp. Fig. 4). Considering the squareness gridness score, we observed similar results indicating a difference in mean gridness for S1 vs. S2 trajectories (p = 51e−10; Two-sample t-test) and distribution comparison (p = 2.5e−14; Two-sample Kolmogorov–Smirnov test). In brief, these results suggested that gridness was present for both trajectories, even though S1 had higher gridness values than S2.

We also analyzed the effect of S1 and S2 trajectories on position estimates only from grid cells activity among the dorsal to ventral modules in each time step. We expected that the regular hexagonal tessellation as in S1 would present more consistent position estimates between the modules than the less clear tessellation as in S2. We computed the variance of the animal’s current position estimates across modules to detect differences between S1 and S2. The variance of position estimates among modules fluctuated less in S1 (Fig. 3C-left) compared to the S2 trajectory (Fig. 3C-middle) throughout the exploration. This difference in variance indicated dissimilar means (p = 1.6e−179; Two-sample *t*-test) and variance levels (p < 1e−200; Two-sample *F*-test for equal variances; Fig. 3C-right) between trajectories; similar results were observed excluding the most ventral module to test whether the variance is driven by that module (p = 9.91e−144; Two-sample *F*-test for equal variances). Furthermore, when computing the absolute (Euclidean) distance error about the animal’s current position estimates across modules, we observed less fluctuation (variance) over time for the S1 compared to the S2 trajectory only for modules 1, 4, and 5 (p = 5.0e−14, p = 3.7e−18, p = 4.7e−48, respectively), but not for modules 2 and 3 (p = 0.32, p = 0.48, respectively; Two-sample *F*-test for equal variances; Fig. 3D). For both S1 and S2, the most ventral module (module 5) differed in error compared to dorsal modules (Fig. 3D). The average variance across modules for S1 was higher than for the S2 (p = 1.9e−47, p = 2.6e−46, p = 4.7e−43, p = 2.5e−136, p < 1e−200; Two-sample *t*-test; Fig. 3D). These observations indicated that the scaling properties of grid cells influenced the estimates, where scaling along the dorsal-to-ventral modules did not seem strictly necessary for optimal error reduction.

These results indicated that the grid tessellation depended on the animal's trajectory. Since in S2 the animal was mostly near the walls, the tessellation was distorted compared to S1 because the trajectory sampling did not cover the arena uniformly. This observation does not convey that when firing fields were less regular (as during the S2 trajectory), the information represented by grid cells is less accurate for estimating the animals’ position as we will see in the next section. Our results just suggested that the grid’s firing fields along the modeled dorsal-to-ventral modules might represent a simple solution by equally contributing to the estimations of grid cells contingent on the animal’s trajectory.

### The grid network reduced the error accumulation using place fields

**Figure 4:**
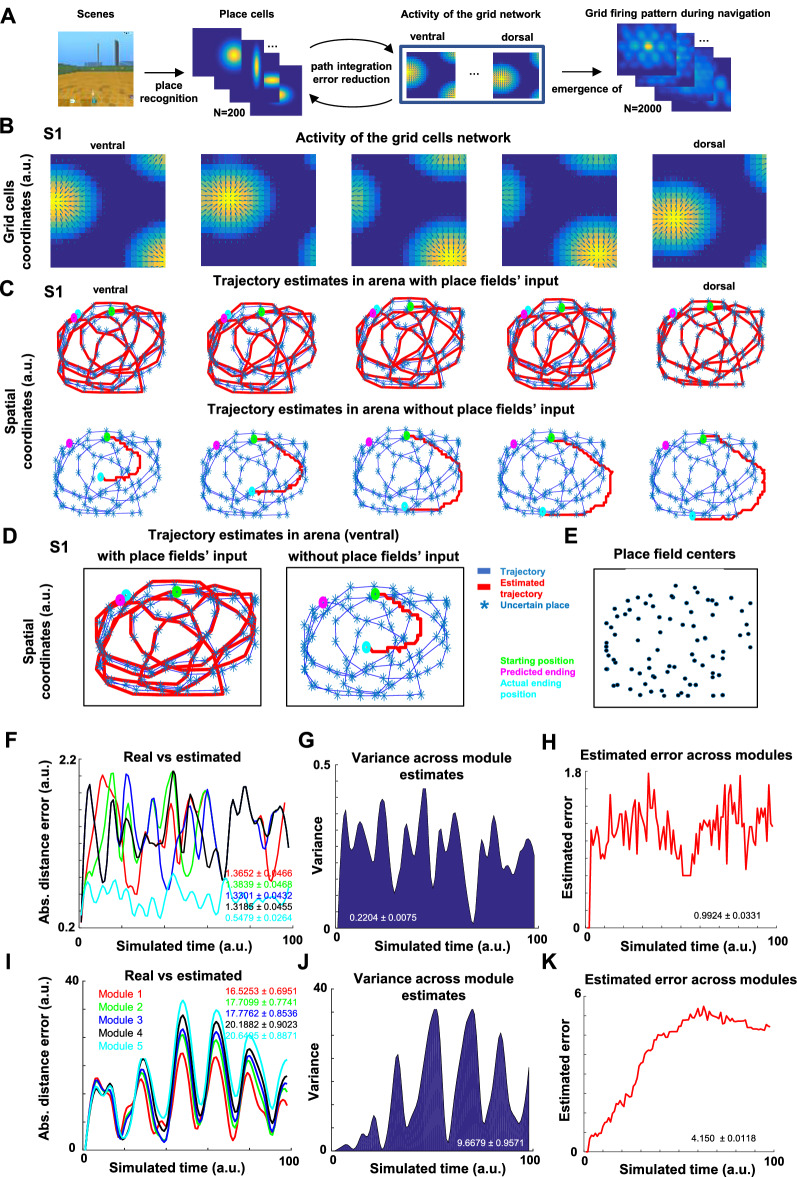
Place field input to grid cells enabled error reduction for path integration. (**A**) Schematics of the place-grid model (further detailed in Suppl. Fig. 8). Scenes obtained from Google DeepMind Lab open source software (https://www.deepmind.com/open-source/deepmind-lab)^61^. (**B**) Example of the “activity packet” (bump) for the grid cell network considering the dorsal to ventral modules individually. The squared dynamical space is represented by the 20 × 20 grid cells. (**C**) Example of the estimated trajectories made by the grid cell modules during an S1 movement across the arena for 1000-time steps at the top row with place field’s input. To evidence that the error accumulation deviates the estimated position from the actual trajectory, the bottom row represents the prediction of each module without place fields’ input for a short trajectory only (100-time steps; red trace) from the starting position (see Supp. Fig. 5 for the whole trajectory). Plots indicate a better prediction of the actual animal’s trajectory when place field inputs are given to grid cells. (**D**) The same trajectories as previously shown for the ventral module were magnified for comparison purposes. The green dot represents the starting position, the magenta dot indicates the predicted trajectory ending, and the pink one is the actual ending position. Asterix represents the places where the activities of grid cells across modules could enable place fields. (**E**) An example of place field centers that emerged during the short trajectory is depicted in panned (**D**). (**F**,**I**) The Euclidean distance error measure compared the estimated and the actual trajectory for dorsal (module 1) to ventral (module 5) modules. Plots indicate that the distance was higher in (**I**) when place field information to the grid cell network was absent. (**G**,**J**) The measure of the variance across estimates showed a similar observation. (**H**,**K**) The estimated error was lower when place field input was provided (**H**) compared to the absence of input (**K**). The inset numbers represent mean ± sem for the associated data for all the panels. All panels except for (**A**) were made using custom code in Matlab R2016b (https://www.mathworks.com/). This work is licensed under a Creative Commons Attribution 4.0 (CC BY 4.0) International License (https://creativecommons.org/licenses/by/4.0/).

The collective activity distribution of grid cells as implemented here had one prominent feature: an “activity packet” (bump) per grid module (i.e., dorsal to ventral) that was driven by the animal’s movement. The dynamical space where this bump moved was defined by a 2D matrix represented by 20 × 20 grid cells for each module (Fig. 4B). When the animal’s velocity changed, the activity packet moved in the grid neuronal dynamic space in a similar proportion. This activity packet and its dynamic move are commonly referred to as “continuous attractor” in the biological and modeling literature^2^. In this scenario, the activity packet created a grid cell’s dynamics that enabled estimating the animal’s position.

We analyzed here whether place field emergence had a role in reducing the error accumulation of grid cells. The presented analyses were based on four assumptions in our model: (I) both place and grid cells were simultaneously active during navigation and represented two different but concurrent neural populations; (II) when place fields emerged in each time step of the simulation, they received inputs from grid cells as explicitly defined for our model (see grid-place cell relationship in the Method section); (III) by computing the position change of the grid cells’ activity bump between consecutive time steps, we intended to estimate animal’s next movement; (IV) inputs from the grid to place cells were implemented based on a diversity of spacings represented by the grid modules (Fig. 3), where place cells feedbacked to grid cells to correct for drift and anchor grids to environmental cues^70^. Regarding the assumption (II), it is worth noting that only the activities of grid cells projecting to place cells varied across the environment. These inputs considered the maximum grid activity per module in each time step and were multiplied by the symmetrical weight matrix to represent the diversity of information provided by the different spacings from the dorsal-to-ventral grid modules. Place fields would only emerge for the active place cells above a certain threshold, given the grid cell input. Note also that place fields were not necessarily defined in the places with above the threshold grid cell input but determined by the place network activities. These properties between grid and place cells remained fixed through all simulations. A simple schematic of the implemented model under these assumptions is shown in Fig. 4A (see also Supp. Fig. 8 for a more detailed explanation).

The activity of the grid network for each module was separately used to compute the spatial position estimates. Emerging place fields affected only the position estimates, not our model's grid cell firing patterns. Figure 4B represents the activity of the grid network across modules that was used to compute the spatial position estimates. Considering the S1 spatial trajectory with and without place cell input to grid cells (Fig. 4C-top and bottom, respectively), the animal’s position was estimated accurately only when place field input was present. In the absence of that input, no grid module  could estimate the animal’s trajectory with precision right from the start position of the simulated animal (Fig. 4C-bottom; Supplemental Fig. 5 shows the whole estimated trajectory). Supplemental Fig. 6 shows similar results but for an S2-like trajectory. Furthermore, Fig. 4D represents the estimated trajectories with and without place field input for the most ventral grid module. The defined place field centers for the animal’s trajectory were plotted in Fig. 4E. Interestingly, place fields that were defined in the places where the input from grid cells was low (i.e., below 1.1 a.u.), were shown as uncertain places in Fig. 4C,D. This observation indicated that the place and grid cells were dynamically defined and depended on the neural activations in each time step. Computing the Euclidean distance between the position estimates and the animal’s current position, the place field input to grid cells (Fig. 4F) resulted in less fluctuation compared to the absence of the input (Fig. 4I)  across modules (p = 1.0e-53, p = 9.0e−52, p = 1.1e−46, p = 2.6e−51, p = 3.9e−56, respectively; Two-sample *F*-test for equal variances). We observed that the most ventral module (module 5) had a different fluctuation than other modules in the presence of the place cell input (Fig. 4F), but not in the absence (Fig. 4I).

**Figure 5:**
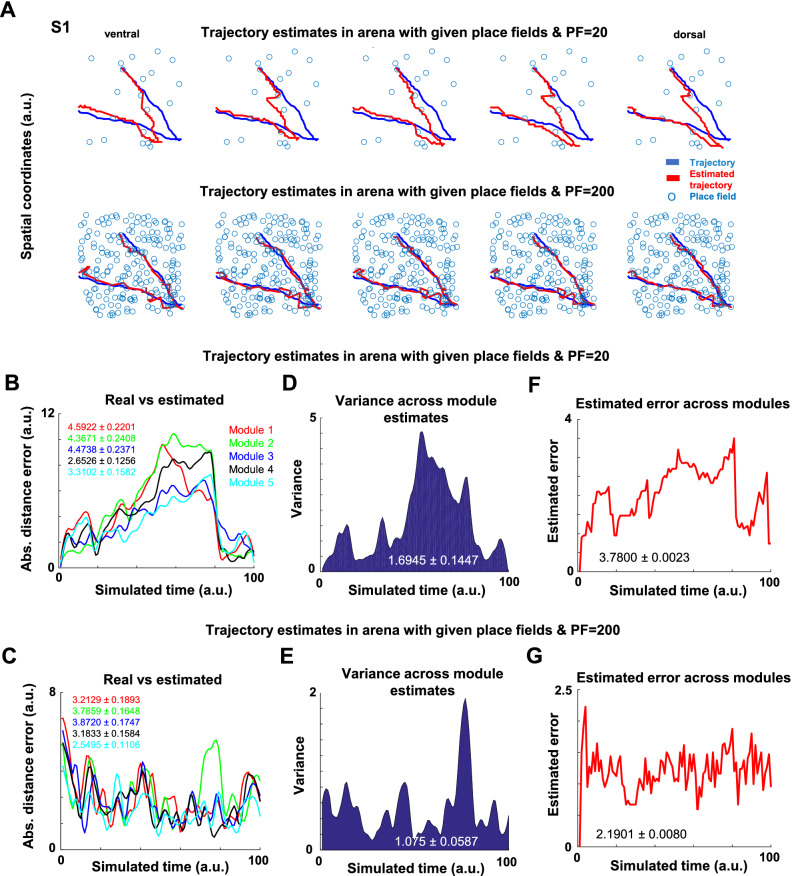
A lower variance and estimated error across grid modules were observed with closer place fields to the animal trajectory. (**A**) A brief trajectory example and the estimated error from the grid cell network when a small (PF = 20) or high (PF = 200) number of place field centers were spread randomly across the arena. Blue lines represent actual trajectories, and red ones indicate trajectory estimates. (**B**,**C**) An example of Euclidean distance error measure shows that (**B**) when place field input was provided and place fields were set to PF = 20, the error was higher than (**C**) when the number of place fields was set to PF = 200. Module 1 refers to dorsal and module 5 to ventral. (**D**,**E**) The measure of variance in fewer place fields (**D**) vs a larger number of place fields (**E**), indicated a higher variance for the former case. (**F**,**G**) Example of the estimated error when (**F**) PF = 20 vs (**G**) when PF = 200 across modules. The inset equations represent mean ± sem for the associated data for all the panels. All panels were made using custom code in Matlab R2016b (https://www.mathworks.com/). This work is licensed under a Creative Commons Attribution 4.0 (CC BY 4.0) International License (https://creativecommons.org/licenses/by/4.0/).

A similar observation was obtained when plotting the variance across modules, indicating different means (p = 7.1e−19; Two-sample *t*-test) and variance levels (p = 8.5e−177; Two-sample *F*-test for equal variances) with or without the place field’s input for S1 (Fig. 4G,J). The estimate indicated a higher mean of error in the absence of place field inputs to the grid cell estimate than in the presence of the input (p = 3.7e−29; Two-sample *t*-test; Fig. 4H,K; see “Methods”). Performing the same analyses for the S2 trajectory presented similar results as for S1 (Supp. Fig. 6). When place field input to grid cells was provided (Supp. Fig. 6B), it resulted in less absolute distance error compared to the absence of the input (Supp. Fig. 6E) across modules (p = 3.9e−43, p = 1.7e−42, p = 4.1e−42, p = 9.2e−44, p = 6.7e−49, respectively; Two-sample *F*-test for equal variances). Plotting the variance across modules in these situations indicated different means (p = 1.5e−24; Two-sample t-test) and variance levels (p = 8.1e−187; Two-sample *F*-test for equal variances) for S1 and S2 trajectories (Supp. Fig. 6C,F). The estimated error showed a higher mean of error in the absence of place field inputs to the grid cell estimate than in the input’s presence (p = 9.2e−46; Two-sample *t*-test; Supp. Fig. 6D,G). These results informed us that  the grid cell network could reduce the error accumulation using the emerged place fields during navigation in our model.

Overall, the rate maps in Fig. 3 with an isolated grid network showed stable grid firing patterns throughout the session. However, our results indicated an intrinsic accumulation of error when estimating animal’s location even without an induced noise source to the grid network. The estimation error is rooted in the grid cells’ connectivity matrix that determined the activity bump (see “Methods”-position estimation). The connectivity matrix affected the position of the activity bump because the matrix represented a discretization of the bump’s movements in the neural space. When estimating  the position of the animal based on the previous position and the magnitude and direction of the bump’s movement, there was an error accumulated in the estimation that depended on where the activity peak was placed in the grid matrix. Consequently, if the activity bump moved to certain coordinates in the matrix at time *t* relative to time *t*-1, then there was a discretization of how many relative translational units within the matrix (i.e., how many positions in the matrix and the direction of that relative movement) we considered determining the magnitude of the animal’s movement.

### Closer place fields increased spatial precision for grid cells’ location estimation drift

We next analyzed whether closer or distal place fields to the animal’s current position differently reduced the error accumulation. To address this issue, the number and the distribution of place fields were manipulated. Their effect on place estimation during a short trajectory was evaluated (Fig. 5). The place fields were randomly placed across the arena during the S1 trajectory under two scenarios: a fewer number of place fields (PF = 20; Fig. 5A-top) and a larger number of place fields (PF = 200; Fig. 5A-bottom). The rationale in proposing this configuration was that the distance between the place fields and the animal’s current position followed a Gaussian distribution in both scenarios, but because of the difference in sampling (i.e., PF = 20 vs. PF = 200) the distances of place fields to the animal’s current position would have been higher in the fewer place fields scenario.

Our model with place field inputs on grid cells showed a good estimate of position across modules only when the number of place fields was high (Fig. 5A-bottom). Specifically, we compared simulations in which there were relatively fewer place fields (Fig. 5B) to those with a larger number of fields (Fig. 5C). Computing the Euclidean distance between the position estimates and the animal’s current position resulted in less fluctuation with a larger number of place fields across modules (p = 3.9e−06; p = 0.05; p = 0.04; p = 0.01; p = 1.1e−04, respectively; Two-sample *F*-test for equal variances). A similar observation was obtained when plotting the variance across modules, indicating different means (p = 1.0e−04; Two-sample *t*-test) and variance levels (p = 2.8e−17; Two-sample *F*-test for equal variances) for S1 and S2 trajectories (Fig. 5D,E). The estimated error for such comparison regarding place field positions indicated a higher error mean when a relatively small number of place fields was provided compared to higher numbers (p = 1.3e−05; Two-sample *t*-test; Fig. 5F,G; see “Methods”).

Overall, these results indicated that when place fields were statistically closer to the actual animal trajectory, there were less variance and error across grid modules. The high number and closer place fields to the animal’s trajectory were the most critical factors in reducing the error. As the number of place cells increased, the possibility of having a field near the animal's trajectory also increased. The evidence was in line with the notion that place fields correct for drift and anchor grids to environmental cues^70^. Our model supported this hypothesis by showing that these defined place fields must be relatively close to the animal’s trajectory.

## Discussions

Several modeling studies in the literature have proposed that place cell inputs can stabilize and reduce accumulated error in grid firing patterns^43,71,72^. Our work goes beyond these observations by examining the influence of dynamic integration of emerging place fields in a new environment, rather than defining static preassigned place fields, reaching similar conclusions to those previous works.

The main contribution of this work is that we characterized how to incorporate place cells-like information into grid path integration for error reduction. We hypothesized that the dynamic coupling between place- and grid-like cells with position information in the form of place fields would have integrated velocity inputs and spatial information in the grid network for path integration. Spatial entropy and information density measures characterized the evolution of place fields’ emergence during exploration. This study effectively demonstrated that place cells could dynamically anchor grid cell activities to reduce the error accumulation in a continuous attractor model. The results showed a simple but realistic representation of place cells allowing a simulated animal to encode spatiotemporal information relative to its visual and distal information for place field definition. The model identified salient features in visual scenes obtained from the 3D DeepMind Lab environment in a realistic fashion^61^. Furthermore, we showed that idiothetic information enabled the grid network to obtain an updated current position estimate. The analyses finally characterized how increasing the number of emerging place cells through their place fields affected grid error reduction.

Our position estimates considered each grid modules' activity separately. We could also have combined grid modules’ activity for decoding, as commonly proposed in empirical studies. It is still uncertain, however, how the brain makes such a combination, given the different grid scales along the dorso-ventral axis. It is also uncertain which brain structure sustains global estimates for position representation. In fact, it has been recently proposed that the coordination of activity across individual modules is necessary to assess how grid cells encode the brain's internal representation of position^73^. In this respect, Waaga et al. indicated that there are specific mechanisms between grid-cell modules, but it is unclear from that work how an overall estimate would emerge from grid modules. Supp. Fig. 7 shows the estimated trajectories using a global trajectory estimate when averaging estimates from all the modules in each time step with and without place fields’ input. It can be seen in Supp. Fig. 7 that even after averaging across modules, the position estimate is accurate only with input from place cells.

### Vision is sufficient to prompt place cell responses to encode local cues

It has been reported that animals recognize landmarks through visual identification, and the animal’s current location relative to a landmark is encoded and remembered^56,74,75^. Within this cognitive framework, coding places is possible even for a simple neural network that processes an image frame in each time step. Previous studies discussed consequently that a complicated cognitive mechanism seems unnecessary^56^. In this respect, the simplicity of a cognitive mechanism was satisfied in our model by two conditions to determine one’s location relative to the landmarks^74^: (a) a function of the feature space that changed among landmarks but did not change with different viewpoints of the same landmark; (b) a function of the feature space that changed with one’s relative position to the landmark. These functions were implicitly embedded in the model described in this work, and the model conveyed enough spatiotemporal information from visual processing to enable dynamic place information; c.f.^76,77^. These sensory neurons provided a complementary but complete representation of the experienced environmental layout, regardless of specific landmark features and boundaries in the simulated environment. Considering our previous works on place field emergence^78,79^, the described simulation showed that vision is sufficient to prompt place cell responses that resemble local cues and other spatial distal cue identification^9,57,80^. However, we cannot conclude whether these cell-like profiles differ in their underlying mechanisms. We can only say that the modeled place cells represented the animal’s position relative to a visually recognized local and a distal landmark.

### Place cells can emerge as a by-product of the experience

Our results agree with the idea of experience dependent place field formation. However, the experience-dependent formation of place fields contradicts evidence for an underlying hardwired circuit in the hippocampus^59,81–85^. In more detail, in^86^ sought to address this issue by recording place fields as rats entered a novel environment. Ten of the 12 recorded cells appeared to have spatial firing fields immediately, indicating that the place-cell map was largely predetermined. Although this evidence suggests the possibility of preexisting maps, experience also has a critical role in shaping hippocampal maps of space^87^. Place fields are expressed in some form when animals are put into an environment for the first time, although the map may evolve further with experience. In^88^ this phenomenon was also shown on a novel arm of a radial maze, in which some place fields only became evident after 1–2 min experience. If place cell formation is emerging from experience instead of being hardcoded in the brain, that would indicate the existence of a specific group of place cells responding solely to spatiotemporal relative information. Verifying this will help us to get a deeper insight into how we perceive and interact with space and time in our daily experiences. Our study suggests that place cell formation can emerge from experience instead of being hardcoded in the brain. This phenomenon presupposed the existence of a specific group of place cells responding solely to spatiotemporal relative information. Our entropy analyses enabled us to characterize aspects that emerge from experiencing the environments. Those emerging aspects were relevant to spatial information based on the shape of place field distribution (i.e., how the population of place fields spread across the environment) and on the number of events (i.e., the number and the spiking activity of place cells). Our analyses also identified a tradeoff between the shape of the distribution and the number of place fields. When the number of place fields increased, the spatial entropy got larger. Nevertheless, the shape of the distribution made a difference. This work suggested that the animal’s movement strategy was helpful for place field development because the network quickly reached high coding complexity and spatial information after early exploration of the animal-like model. The place cells processed plain visual features as anchoring points, allowing navigation to proceed in environments with a few free-standing landmarks. We saw a complex tradeoff between the density and the number of events that emerge through experience.

### There are still open questions on the place-grid cell relationship

We showed that a combination of idiothetic and allocentric sensory inputs enabled error reduction in grid cells through place field information. Experimental observations contradict the assumption of place cells taking a role in incremental (and relatively slow) spatial coding^89^. Furthermore, some theoretical models commonly define fixed place fields during the training of grid networks from the beginning of simulations, disregarding the dynamics of place cell-like emergence^57,76,77,90^. Still, recent works have revealed that grid cells are modulated by environmental changes commonly associated with place cells’ detection, suggesting a more interconnected representational mechanism between internally generated path integration signals and sensory processing^91,92^. In this, further studies should evaluate the effect of place field size (see Fig. 1E,F) on the grid cells’ error reduction for path integration. We expect that smaller place fields would produce a better error reduction than wider fields.

Our work proposed that allocentric place cells (e.g., based on vision) can be used to recalibrate the activity of the grid cells in case of path integration errors, and, in turn, the activity of grid cells is used to enable place cells to disambiguate between two visually similar places^15^. Our observations align with the experimental evidence indicating that a toroidal topology of grid cell populations tends to accumulate error over time^93^. Our model implied that grid cells could exist without place field input, which has some parallelism with experimental data showing that grid cells maintain synchrony during hippocampal inactivation even when the grid pattern is lost^94^. This experimental evidence showed that grid cells require an excitatory drive from the hippocampus as one prerequisite for forming and translocating grid patterns in the MEC^27^. Consequently, we can infer that when the grid pattern is lost, the grid cells cannot estimate the animal’s position in biological networks. However, further experimental evidence is needed to prove our observation.

## Methods

All data analyses and scripting reported in this manuscript were made through custom code using Matlab R2016b. This work is licensed under a Creative Commons Attribution 4.0 (CC BY 4.0) International License (https://creativecommons.org/licenses/by/4.0/).

### Visual processing

#### Image collection

We collected over 100,000 full-color images (~ 40 images per second) of a square simulated arena using the 3D Google DeepMind Lab simulator open source software (https://www.deepmind.com/open-source/deepmind-lab)^61^ taken from a simulated atop camera on a simulated, rodent-like animal (i.e., a mobile robotic agent). The images referred to partially observed and visually diverse landmarks. Each experiment reported in this work enabled the animal to explore and quickly interact with the environment. Images of 84 × 84 pixels with a bin depth of 24 were obtained from multiple viewpoints depending on the animal’s movements. Because the image set had a certain redundancy of scenes (i.e., images taken with the same orientation in nearby places), the dataset processed in each experiment was a down sample of all the image sets taking every 40 images starting with the first.

#### Blob detection

Visual blobs were detected from each visual scene (image frame) using the algorithm reported in^95^ (image processing module from Blob Detection Toolbox—Imperial College London). We defined visual blobs as a set of pixels after three main steps: (i) toboggan edge-preserving smoothing; (ii) applying an interest operator which was designed to be scale and color invariant; (iii) employing morphological operators and connected component analysis to extract convex regions, which did not constitute straight lines and occupied a considerable amount of the whole scene. There was no restriction on the number of blobs detected in an image to ensure the representation of the entire scene. The images themselves did not comprise far fewer regions of approximately uniform intensity. Without merging blobs, multiple blobs occupying part of the area of a particular region with uniform intensity did compete. By intermittently saving the state of each blob at each time step of the simulation, the algorithm created a database of blobs at varying sensitivity levels to intensity changes. To use blob information for matching the place cell’s preferences, the algorithm detected the area of each blob, the angle of orientation of the central axis, solidity, and centroid coordinates in the image. Few other simple features such as average intensity, elevation, azimuth (i.e., the vertical and horizontal positions of the blob center in the image, respectively), size, eccentricity (i.e., the ratio of the lengths of the major and minor axes) were not included but measured for future investigations.

### Experimental definition

#### Environmental configuration

We set up a Google DeepMind Lab environment for each experiment to obtain visual scenes and recorded the positional statistics of the simulated animal in each time step. For the most straightforward configuration (i.e., search and avoid factory in a squared arena), a square arena with a side length of $L=100$ (dimensions: 100 × 100 ×  + ∞ arbitrary units) was defined. At the beginning of the simulation, the animal was placed in the center of the arena with a random orientation, leaving the animal to explore the arena freely. Despite the animal moving in 2D space, the six borders in a 3D space were regarded as landmarks. The animal perceived environmental cues using its visual (simulated atop camera as previously indicated) or distance sensing, then processed the information through its place cell network.

### The place cell model

#### Proximity sensor model

Considering the 3D simulated environment without any references other than the delimiting walls of the arena, the simulated animal only got visual or proximity information from walls or distal landmarks placed further than the walls (landmarks). To study the locating function of place cells, we referred to that egocentric information of position as front (F), back (B), left (L), right (R), up (U), and down (D) in analogy to^62^. The distance up and down was not considered in the model and was kept constant. In this way, we naturally approached the referencing place in animals (c.f., 2D reference in^64^). The actual location of the animal at time $t$ was described, however, based on just L-R, B-F, D-U main axis information. This part was made through the actual distances for the 2D space to walls in a vector representation $X\left(t\right)$= (${x}_{i}\left(t\right)$, ${y}_{i}\left(t\right)$, ${z}_{i}\left(t\right)$), were ${z}_{i}$ is kept constant. Because it was assumed here that the acquired perception should not be accurate due to sensory noise of its locations, then the input in each coordinate added an error of sensory perception as ${X}^{{\prime}}\left(t\right)=\left({x}_{i}\left(t\right), {y}_{i}\left(t\right), {z}_{i}\left(t\right)\right).(1+\alpha \eta )$, where $\alpha$ represented the error rate of the sensory (visual or proximity) perception. The random number, $\eta$, was taken from a uniform distribution within the interval [− 1, 1], that is $\eta \sim U(-\mathrm{1,1})$^63^. That error ${X}^{{\prime}}\left(t\right)$ conveyed to place cells resembling how boundary vector neurons represent the distance to the animal^65^, which affected the firing of place cells^96^. A feed-forward network was implemented to determine the locating system constituted of place and sensors^63,64,96^.

#### Energy consumption

To compute the firing profile of the place cell network, the implemented model was based on^55,62^, calculating the energy consumption of a place cell through the Hodgkin-Huxley (H–H) definition. The neural energy was measured as:

$${C}_{m}\frac{{dV}_{m}}{dt}={g}_{l}\left({E}_{l}-{V}_{m}\right)+{g}_{Na}{m}^{3}h\left({E}_{na}-{V}_{m}\right)+{g}_{k}{n}^{4}\left({E}_{k}-{V}_{m}\right)+I$$

$$\frac{dn}{dt}={\alpha }_{n}\left(1-n\right)-{\beta }_{n}n \frac{dm}{dt}={\alpha }_{m}\left(1-m\right)-{\beta }_{m}m \frac{dh}{dt}={\alpha }_{h}\left(1-h\right)-{\beta }_{h}h$$

$${\alpha }_{n}=\frac{0.01(10+{V}_{m}-{V}_{r})}{exp[((10+{V}_{m}-{V}_{r})/10)-1]}{\beta }_{n}=0.125 exp \left(\frac{{(V}_{m}-{V}_{r})}{80} \right)$$

$${\alpha }_{m}=\frac{0.1(25+{V}_{m}-{V}_{r})}{exp[((25+{V}_{m}-{V}_{r})/10)-1]}{\beta }_{m}=4 exp \left(\frac{{(V}_{m}-{V}_{r})}{18} \right)$$

$${\alpha }_{h}=0.07 exp{((V}_{m}-{V}_{r})/20){\beta }_{h}=\frac{1}{exp[((30+{V}_{m}-{V}_{r})/10)+1]}{\beta }_{m}$$

These terms represented the neuron components as follows: ${V}_{r}=-65 \; \text{mV}$ as the resting membrane potential; $m=0.5$, $h=0.06$, $n=0.5$; $I = 10$; the input current as 0.1 mA; $bas{e}_{V}= 65$; three different ion currents: ${g}_{Na}$= 120 maximum Na +  conductance mS/cm^2^; ${e}_{Na}$= 150 mV indicating the Nernst potentials of Na +; ${g}_{k}$= 36 indicated the maximum K + conductance mS/cm^2^; ${E}_{k}=-12 \;\text{mV}$ represented the Nernst potentials of K +; ${g}_{l}$ = 0.3 was the leakage conductance mS/cm^2^; ${E}_{l}= 10.6 \, \text{mV}$ as Nernst potentials while there is no leakage current; $dt=0.01$ time step for forward Euler method. The additional ‘gating’ variables $m$, $n$, $h$ model the probability that a channel is open at a given moment in time. The combined action of h controls the Na+ channels while the K + gates are controlled by $n$^97^. The specified values were used for the simulation parameters 1 of the H–H model. For simulation parameters 2, the number of place cells was as set to ${N}_{pc}=2000$; Number of proximity sensors was ${N}_{s}=3$; Sensory error rate as $a=0.1$; Learning rate as $\mu =0.001$; Maximum firing rate as ${R}_{m}=20\;\text{Hz}$; Firing threshold as ${P}_{thr}=0.3$; Number of simulation steps sets to 10,000; Step length as 1.

#### Place cell learning rule

A feed-forward network was defined as fully connected, linking proximity sensors (${N}_{s}=3$) and place cells (${N}_{pc}= 2000$) through a synaptic-like weights matrix $W(t)$, with ${w}_{ij}(t)$ the connection weight from the $i$-th sensor to the $j$-th place cell at time t. Based on^96^, weights were initialized as ${w}_{ij}(t)$ = ${(1+exp((\gamma -E(\gamma ))/2{\sigma }^{2} ))}^{-1}$, with $\gamma \sim U(\mathrm{0,1}),$ and expectation $E(\gamma )=0.5$. Using that definition of ${w}_{ij}(t)$, we imposed that place fields could emerge both near and far from the boundary of the environment them; c.f.,^96^. Weights were the basis vectors in the model, which were used to compute the firing powers of place cells. When the competitive learning rule was employed, place cells became tuned to a specific input, which led to the spatial selectivity^62^. Using the firing rate model proposed in^64,65^, we defined the firing power ${P}_{j}^{f}(t)$ of the $j$-th place cell at time $t$ as: ${P}_{j}^{f}\left(t\right)=C{R}_{m} exp(-{(1/n \| X{^{\prime}} (t)/L-{w}_{j}(t)\| )}^{2}/(2{\sigma }_{j}^{2}))$, where ${R}_{m}$ is the maximum firing rate of a single place cell, which is about 20 Hz. The norm represents the Euclidean distance, $n$ is the number of sensory inputs, and ${w}_{j}\left(t\right)$ is the $j$-th row of $W(t).$ $C$ is the energy consumption by a place cell during an action potential which was ~ 188 nJ (Suppl. Fig. 1—see “Methods”—H–H model simulation parameters 1) as the energy consumed to transmit a spike as previously described; $C$ was normally distributed from $N(\mathrm{188,10})$ nJ, and ${\sigma }_{j}$~ $N(\mathrm{0.03,0.005})$ both reflecting the diversity of place cells’ metabolic environment^62^. The learning rule was applied not only in a batch manner^63^ but also on an energy level as follows: $d{W}_{j}(t)/dt=\mu (X{^{\prime}} (t)/L-{W}_{j}(t))$ with the responding set $J= \{j|{P}_{j}^{f}(t)> {P}_{thr}^{f} \}$. The learning rate was defined as $\mu$ and ${P}_{thr}^{f}$ was the minimum firing power of neurons that are activated. Consequently, every place cell responding to the current location with a firing power above the threshold (together with the feature matching criteria explained beneath) did modify the weights from sensors.

#### Spatial location

As indicated in^62^, the spatial location $X\left(t\right)$ can be seen as a function of the firing power of place cell $j$. Then the place field of neuron $j$ in our model was defined as the set of all the locations $X\left(t\right)$ with firing power larger than ${P}_{thr}^{f}$. The field centers were determined from the positions within the corresponding place fields. The center of the place field associated with the $j$-th place cell during this dynamical process is ${C}_{J}={\int }_{0}^{+\infty } {P}_{j}^{f}\left(t\right){X}^{{\prime}}\left(t\right)dt/{\int }_{0}^{+\infty } {P}_{j}^{f}\left(t\right)dt$. The location of the animal was estimated by the weighted average of place field centers according to the response set as the complement of $Loc\left(t\right)={\sum }_{J} {P}_{j}^{f}\left(t\right){C}_{j}/{\sum }_{J} {P}_{j}^{f}\left(t\right)$ with $J= \left\{j|{P}_{j}^{f}(t)> {P}_{thr}^{f}\right\}$, where the place field was defined as ${C}_{j}$ and ${P}_{j}^{f}\left(t\right)$ was the activity power of the $jth$ neuron at moment $t$.

#### The feature matching model

Place cells matched features from the sensed environment as in^56^. Each place cell was tuned randomly to detect two visual blobs describing four features: area, orientation, solidity and centroid coordinates. Each place cell was therefore represented by two sets of feature values, M1 and M2. This pair of feature values modeled the neuron’s preference that would maximize the place activation. The similarity of a place cell’s ideal feature value, ${\mu }_{f}$, to the corresponding feature value of a blob ${x}_{f}$ was measured using a Gaussian function: ${G(x}_{f},{\mu }_{f},{\sigma }_{f})={e}^{{-({(x}_{f}-{\mu }_{f})/{\sigma }_{f})}^{2}}$. When ${x}_{f}$ and ${\mu }_{f}$ were close, $G$ tended to 1; as their difference increased, the function approached zero. The magnitude of ${\sigma }_{f}$ determined the sensitivity of the function to the difference between ${x}_{f}$ and ${\sigma }_{f}$, which was set to 0.5 in this work. The response of a place cell to a particular blob ${B}_{i}$ was determined by the product of feature similarities over the set of features $F$: ${A}_{Bi}={\prod }_{f\in F} {G(x}_{f},{\mu }_{f},{\sigma }_{f})$. All features of ${B}_{i}$ did have to be like the place cell’s maximal response value for a high activation. In that case, the place cell responded to conjunctions of features. Note that there was no predetermination of which blobs were to be associated with which place cell. Those visual blobs ${B}_{i}$ and ${B}_{j}$ that generated the strongest response to the two sets of place cell feature values M1 and M2 determined the activation of the place cell: $A={{max}_{i\in B}A}_{Bi} . {{max}_{j\in B,j\ne i}A}_{Bj}.$ Note that from one simulation to another, the same modeled place cell would show different place fields, because the neuron’s preference for blob detection and other parameters were randomized at the beginning of each simulation.

#### Place field center adaptation

The modeled place cells were initially tuned to random feature values, which were unlikely to correspond to any environmental blob. Therefore, place fields were initially quite broad and weak, conveying little spatial information. To increase the spatial information content, a competitive learning algorithm was used to adapt each place cell's feature value ${\mu }_{f}$ to the corresponding blob feature value ${x}_{f}$. For each iteration $t$ in the competitive algorithm, the place cell whose activation was the highest at a particular position was determined to be the ‘winner’ of that position. For each position, the pair of visual blobs that maximized the activation of the winning place cell were used to train the feature values of the winning place cell. Each feature value of the winning place cell was adapted to the corresponding blob feature value according to the following equation: ${\mu }_{f}^{t+1}=$ $\alpha {x}_{f}+(1+\alpha ) {\mu }_{f}^{t}$. The coefficient $\alpha$ controled the rate at which feature values were adapted. For our simulations, the α value of 0.5 was used.

### The grid cell model

#### Continuous attractor network (CAN)

Theoretical works^17,58^ presented evidence that grid cells can be modeled through toroidal representations and as a symmetric locally connected neural network^71^ which has been recently confirmed experimentally^98^. The model of grid cells that we implemented was based on a twisted torus topology formed by neurons and weighted connections between them. Excitatory and inhibitory connections were defined between nodes to obtain local cooperation and distal inhibition^15,17,57^. This caused neurons that were closer together to become mutually excited, increasing their activity, while the activity of distant neurons got inhibited. The model dynamics caused an “activity packet” (bump) that was modified by the input to the neurons. The shape of the bump was modified by the neurons’ weights, while the localization of the activity in the network was guided by external inputs (i.e., the velocity information of the animal’s movements). The model was implemented in Matlab R2016b based on $N$ = 20 neurons organized in a rectangular matrix, such that $N = {N}_{x} x {N}_{y}$ (with $N=400; {N}_{x}=20; {N}_{y}=20$) represented the repetitive structure of grid subfields. The synapses connect neuron $i$ with $j$, where $i$, $j$ $\in \{\mathrm{1,2}, \dots , N\}$, were defined by Gaussian weighted function. The connection between neuron $i$ and $j$, are ${w}_{ij}={I}_{ai} exp \left(-{\| {c}_{i}-{c}_{j}\| }^{2}/{\sigma }^{2}\right) -T$, where ${I}_{ai}$ was defined as the activation intensity parameter, $T$ was the shift parameter affecting the inhibitory-excitatory balance, σ regulated the size of the Gaussian, and ${c}_{l}$ defined the position of the neuron as represented by ${c}_{l}=\left({{c}_{l}}^{x}-{{c}_{l}}^{y}\right)$, with ${{c}_{l}}^{x}={(l}_{x}-0.5)/{N}_{x}$ and ${{c}_{l}}^{y}={\sqrt{3/2} (l}_{y}-0.5)/{N}_{x}$, where ${l}_{x}$ and ${l}_{y}$ were the column and row of neuron $l$. The dynamics of the grid cells utilized in this work were governed by ${A}_{i}\left(t+1\right)=f({B}_{j}\left(t+1\right)+\tau ({B}_{j}\left(t+1\right)/\overline{{B}_{j}\left(t+1\right)}-{B}_{j}\left(t+1\right)))$, where ${A}_{i}$ represented the activity level of the neuron $i$, with $i\in \{\mathrm{1,2}, \dots , N\}$, $\tau =0.95$ was defined as the parameter that determined the stabilization strength, and $f$ as a simple rectification, non-linear function such that $f\left(x\right)=x$ for $x>0$ and was 0 otherwise. The activity of the neurons depended on the transfer function ${B}_{j}\left(t+1\right)={\sum }_{i=1}^{N} {A}_{i}\left(t\right){ w}_{ij}\left(t\right)$, where ${w}_{ij}\left(t\right)$ represented the weight connecting neuron $i$ and $j$. The network was initialized with a random activity using a uniform distribution between 0 and $1/\sqrt{N}$. The position estimation depended on the imputed planar velocity that subsequently was integrated to obtain a position estimation. The network took as input a modulated version of the planar velocity defined as $v\left(t\right)={[{v}_{x}\left(t\right),{v}_{y}(t)]}^{T}$ where ${v}_{x}\left(t\right)$ and ${v}_{y}(t)$ were the velocities in the $x$ and $y$ direction at time $t$. The input to the network was then modulated by a gain parameter and a rotation matrix such that ${v}^{R}\left(t\right)=\alpha {R}_{\beta } v(t)$, where ${R}_{\beta }$ defined a rotation matrix that depended on the bias angle $\beta =0$ and the gain $\alpha \in + real numbers$ (with $\alpha =1$). Here the vector $v$ did not carry any information about the current position of the animal. The grid remained stable when no velocity input was introduced (i.e., $v=[0; 0]$). However, when the animal moved, this affected the activity of the network shifting the bumps. The effect of the velocity in the network was introduced in the synaptic weight equation as ${w}_{ij}={I}_{ai} exp \left(-{\| {c}_{i}-{c}_{j}+{v}^{R}\left(t\right)\| }^{2}/{\sigma }^{2}\right)-T$, where $\| \|$ represents the Euclidean norm. The grid network was parameterized to model different scale increasing from dorsal to ventral module spacing (0.95) of the grid, and gains as [0.04, 0.05, 0.06, 0.07, 0.08]; the threshold torus equal to 5^17^. With higher gain values, we got denser grids (and therefore smaller spacing between grid subfields), whereas higher bias values rotated the grids^15,58^.

#### Position estimation

The activity of the grid cells (i.e., the bump activity) was used to obtain an updated estimation of the animal’s position. Considering at ${t}_{0}$ the estimated position $pos\left({t}_{0}\right)=[0; 0]$, it was updated as $pos(t + 1) = pos(t)+\gamma \Delta A$ in every time step, where $\Delta A$ defined the change in activity bump (i.e., the magnitude and direction of the bump’s movement). This update was individually made by the network of grid cells in each grid module; $\gamma$ was a parameter that regulated the grid spacing. Varying the spacing and the orientation of the grids by changing the gain and bias parameters, we tested whether there was a difference in module place estimates (see the main text). To determine whether a correction of the estimated position was made in every time step of the simulation, an estimated error was computed as follows: ${E}_{error}=\sqrt{{std({pos}_{x})}^{2}+{std({pos}_{y})}^{2}}$, where ${pos}_{x}$ and ${pos}_{y}$ represented vectors of the estimated animal’s position along grid modules in both spatial coordinates, respectively. If the estimated error ${E}_{error}$ was higher than a predefined threshold (i.e., 1.5 a.u.), the animal’s current position represented an ‘uncertain’ place indicating that the grid modules did not reach enough precision to estimate the actual animal’s position. This situation enabled a place cell’s field to emerge on that position only if a place cell was active and the grid modules provided enough input to place cells as previously explained. The correction of the position estimate $pos(t+1)$ was made based on the nearest place field center from the animal’s current position. It was taken 2.5 a.u. as the minimum distance to select a near a place field as a reference point to correct the position estimate. The rationale for using such a correction is that it was previously reported ‘resetting’ at grid network level. This understanding was discussed in grid cells’ activity to the agent’s correct location^20^. It was also discussed that the dynamics between place and grid cells could have a role through feedback projections from the hippocampus to grid cells, anchoring grid cells’ activity to specific spatial locations, thereby resetting the accumulated error to the ground truth^58^. There is experimental evidence supporting this view^27^. In^89^ it was observed that place cells’ firing could shift toward goal locations, and while the grid cells maintain information overnight, the place cells can reset.

#### Grid-place cell relationship

The connection from grid to place cells was implemented through the diversity of spacings from the dorsal-to-ventral grid modules^39^ (Suppl. Fig. 8). This connectivity was implemented based on a symmetrical weight matrix of a Gaussian curve function for each module. Each Gaussian was distributed evenly in the [0;10] range. The maximum grid activity per module was computed in each time step and multiplied by the symmetrical weight matrix to represent the diversity of information provided by the spacings from the dorsal-to-ventral grid modules. After multiplication, the mean value across neurons of the resulting matrix was computed to determine the place cells to be evaluated. The grid input enabled the emergence of place fields for firing place cells on the animal’s current position with a neural activity above 1.1 a.u. (Supp. Fig. 8). Place fields were not necessarily defined in the places where the input from grid cells was high (i.e., above 1.1 a.u.) but were determined by the place network activities. For active place cells, a plasticity rule was computed as ${W}_{G\_P}\left(t+1\right)= {W}_{G\_P}\left(t\right)+\mu *({X}^{{{\prime}}}\left(t\right)-{W}_{G\_P}\left(t\right))$, where $\mu =0.001$ represented the learning rate, and ${X}^{{{\prime}}}$ was the error of sensory perception as previously described. Regarding the inputs from place cells to grid cells, this information was possible in estimating the animal’s current position (i.e., path integration error reduction as indicated in Fig. 4A). Because there is no evidence suggesting that the MEC (or even the hippocampal formation) provides movement-related signals to motor areas, we did not control the motor activities of our simulated animal. Instead, we only used the neural activities to estimate the  animal’s position. The estimate was made from the network’s stable activity bump after computing the path integration from the network that was updated based on the animal’s movements.

#### Gridness measure

To test the hexagonality of grid cells, we used a hexagonal gridness score of the spatial fields based on^69^ and^11^. The score was calculated from a cropped ring of their autocorrelogram considering the six maxima points closest to the center. The ring was rotated 30 degrees per rotation starting from 30 to 150 degrees. For the six rotated angle, the Pearson correlation $C$ was obtained considering the original un-rotated map. Combining as follows the correlations for these specific rotation angles, the final gridness score was^47^: $gridness=1/2 *\left({C}_{60}+{C}_{120}\right)- 1/3 *({C}_{30}+{C}_{90}+ {C}_{150})$. Following descriptions in^69^, we also computed a squareness gridness score to evaluate how square-like grid autocorrelograms were spatially defined. This measure was computed by rotating the autocorrelogram 45 degrees for every iteration to reach angles of 45, 90 ,135 degrees. This score was calculated as: $square \; gridness= {C}_{90}- 1/2*({C}_{45}+ {C}_{135})$.

#### Definition of grid modules

Previous works^8,19,99^ have shown that regular triangular grids can be observed from dorsal to ventral MEC, where the spacing of the grid increases isometrically along the dorsoventral axis. Firing fields of grid cells located at dorsal-most MEC represent small and highly tuned locations, while ventral-most cells display broader and less location-specific firing activity. Based on a previously defined grid cell model^15^, we used the orientation, the phase of the grid, the spacing (minimal inter-subfields distance), and the size of its subfields in our model to represent the topographical organization of MEC. These settings controlled the spacing and the orientation of the grids across modules in the model. We based the dynamics of a population of neighboring grid cells as observed in dorsal-to-ventral MEC, whose grids share the same orientation and spacing, but have different phases^15^.

### Statistical measures

#### Entropy

The proportion of place cells showing activity at a specific space and time was computed as ${p}_{i}={P}_{i}/P,$ with ${p}_{i} \in \left[0;1\right]$ the occurrence of an event $i$ that varied concerning to size ${x}_{i}$, and ${\sum }_{i=1}^{n} {p}_{i}=1$ with $n$ (the number of place cells) indicating the range of probable events. This probability was based on population densities of place cells which represented the chance to observe spiking activity by a place cell at the animal’s current position (i.e., probability of locating a spike in one place cell). If an event occurred (i.e., the spiking activity of a place cell), then the information gained (i.e., the coding of that place in the population of place cells) varied inversely with the size of the probability. When the probability of the event was minimal and the event occurred, then the information gained was high compared to a situation where the probability was very likely. In the extreme case, where the probability of the event occurring was 1, then no information was gained. In general, it is assumed that the raw information gained varied as ${1/p}_{i}$, but the best form of this function needs to be determined for other criteria. In addition, based on the expected value $H$, the overall information for $n$ events was defined as: $H=H\left(n\right)=-{\sum }_{i=1}^{n} {p}_{i}log({p}_{i})$, which is Shannon's information^100^, and it is equivalent to the Boltzmann–Gibbs formula for entropy, where $H \in [0; log(n)]$. When $H=0$, then one event dominated (i.e., ${p}_{k}$ = 1, $\forall {p}_{k}$ = 0, $i\ne k$ and $H=log(n)$, then ${p}_{k}=1/n$, $\forall i$). Thus, the entropy was maximum when the probabilities were all equal and $H=log(n)$. If all the events happened at the same location $x$, the information gained was zero when a new event occurred. Once the population distribution of place fields was uniformly spread across many locations ($\forall {x}_{i})$, and an event occurred, this event gave complete information. There was a trade-off between the spread of the probability distribution across spatial and the number of events. In brief, the newer places were visited in the arena, the more complex and more extensive the internal map represented by place cells was characterized by more and more events. Furthermore, the increase of events did change the shape of the representation, which led to an increase or decrease in information. This trade-off between spatial density and the number of events can be represented by $H$ as an index of complexity. In short, it was assumed that as the exploration of the environment extended, the complexity in terms of information increased. However, this criterion was purely based on the number of their place fields, and there may be strong ordering effects that discount this increase. Entropy calculations also presupposed the additive independence of events^66^.

#### Entropy ratio

In the case of $H$, and to get a measure of relative information, a primary probability distribution acted as a prior since the information was relative to what occurred in which the event sequence is unknown. Thus, to characterize relative information given the prior information, it was defined the entropy ratio as $r=H/{H}_{max}=-{{\Sigma }_{i} p}_{i}log\left({p}_{i}\right)/log(n)$. By normalizing $H$ concerning the maximum value ${H}_{max}= log \left(n\right)$ it was possible to represent a uniform distribution.

#### Complexity ratio

$R$ Represented the complexity ratio based on the complement of the entropy ratio $R=1-r$, which can be written as: ${(H}_{max}-H)/{H}_{max}=I/{H}_{max}$, indicating the percentage of information that the system could achieve by adjusting itself to the most probable state (i.e., a uniform distribution). This measure represented the relative information as information difference $I$. Assuming ${q}_{i}=1/n$ and ${{\Sigma }_{i} q}_{i}=1$, information can be written following^66^ as: $I={H}_{max}-H=-{{\Sigma }_{i} p}_{i}log({p}_{i}/(1/n))=-{{\Sigma }_{i} p}_{i}log({p}_{i}/{q}_{i})$, which clarify the role of the number of events or place fields.

#### Spatial entropy

The complexity ratio tended to discount the effect as the system changed through more events because information formulas measured relative change^66^. Consequently, we can consider probability densities to discriminate the distribution density from the number of events. If the measured area increased (i.e., the animal explored more of the arena), it was expected that the probability over that area also increased, and then the density changed. Thus, by defining an approximation to the density over an area $\Delta {x}_{i}$, there was computed the total area of the system $X$ as ${\sum }_{i} \Delta {x}_{i}=X$. The density can be written as $p\left({x}_{i}\right)={p}_{i}/\Delta {x}_{i}$ or ${p}_{i}=p\left({x}_{i}\right)\Delta {x}_{i}$, and it was assumed that in the limit, the equation converged to the probability density $p\left(x\right)$ as $p\left(x\right)={p}_{i}/\Delta {x}_{i}$. After few steps, the entropy formula $H$ in probability density terms can be represented as the discrete form of entropy $H=-{\sum }_{i} {p}_{i} log \left({p}_{i}/\Delta {x}_{i}\right)-{\sum }_{i} {p}_{i} log \left(\Delta {x}_{i}\right)=S+Z$, with $H \in [0;log(n)]$, where $S$ is the approximation to the continuous entropy. The term $Z$ is the approximation to the information associated with the sizes of the events. $S$ is the formula that was called 'spatial entropy'. This $H$ is composed of $S$ and the 'information density' $Z$—the amount of information represented by the place cell space. In short, when we examined $H$, we did this for the numerical co-variation of its elements $S$ and $Z$. The size of the place fields was kept fix across simulations in $\Delta x$ = 20 a.u.

#### Varying spatial information

To examine changes in the number of events and their density, we did consider the distribution ${q}_{i}$, which normalizes the distribution of the visited places to sum to unity^66^, and form the expected value of its logarithm $H(p)$. Compared to the entropy of the distribution itself $H\left(q\right)$, where these measures were defined as $H(p)=-{\sum }_{i} {p}_{i} log \left({q}_{i}\right)$ and $H(q)=-{\sum }_{i} {q}_{i} log \left({q}_{i}\right) H\left(p\right)$, where $H(p)$ represents the evenness of the density distribution, while $H(q)$ provides the same but for the density. Note that this is a measure of difference indicating how the place field was represented. Based on^101^ we can have: $H(p/q)=H(q)$-$H\left(p\right) = {\sum }_{i} {p}_{i} log \left({q}_{i}\right)-{\sum }_{i} {q}_{i} log \left({q}_{i}\right)$, which resembles the previously introduced equation for the generic information measure in its classical form. Consequently, we can expect that $H(p/q)$ co-vary with the information difference $I={\sum }_{i} {p}_{i} log \left({p}_{i}\right)-{\sum }_{i} {p}_{i} log \left({q}_{i}\right)$^66^. The difference in absolute terms became greater as the difference between the distributions of observations and the size of the environment increased. To this end, computing complexity on information from the animal’s navigation in each time step, we analyzed the relationship between the number of place fields $n$ and the density of population ${p}_{i}$/$\Delta {x}_{i}$.

## Supplementary Information

Supplementary Figures.

## Data Availability

Datasets supporting the findings of this paper are available upon request from the corresponding author.

## Code availability

Custom code used in this paper is available upon reasonable request from the corresponding author.
