# Supplementary material for: Place cells dynamically refine grid cell activities to reduce error accumulation during path integration in a continuous attractor model
Source: Sci Rep. 2022 Dec 12;12:21443. doi: 10.1038/s41598-022-25863-2 (PMC9744848; doi:10.1038/s41598-022-25863-2)
Supplement: Supplementary file 1 — Supplementary Figures. [file 41598_2022_25863_MOESM1_ESM.docx]

**Place cells dynamically refine grid cell activities to reduce error accumulation during path integration in a continuous attractor model**

Jose A. Fernandez-Leon^1,2,3,4*^, Ahmet Kerim Uysal^1^, Daoyun Ji^1^

^1^ Dept. of Neuroscience, Baylor College of Medicine, Houston, Texas, United States

^2^ Universidad Nacional del Centro de la Provincia de Buenos Aires (UNCPBA), Facultad de Ciencias Exactas, INTIA, Tandil, Buenos Aires, Argentina

^3^ CIFICEN, UNCPBA-CICPBA-CONICET, Tandil, Argentina

^4^ Consejo Nacional de Investigaciones Científicas y Técnicas (CONICET), Argentina

* Corresponding author: Jose A. Fernandez-Leon Fellenz (jafernandez@exa.unicen.edu.ar; jafphd@gmail.com)

**SUPPLEMENTAL INFORMATION**

**Supplemental Figure 1**

**
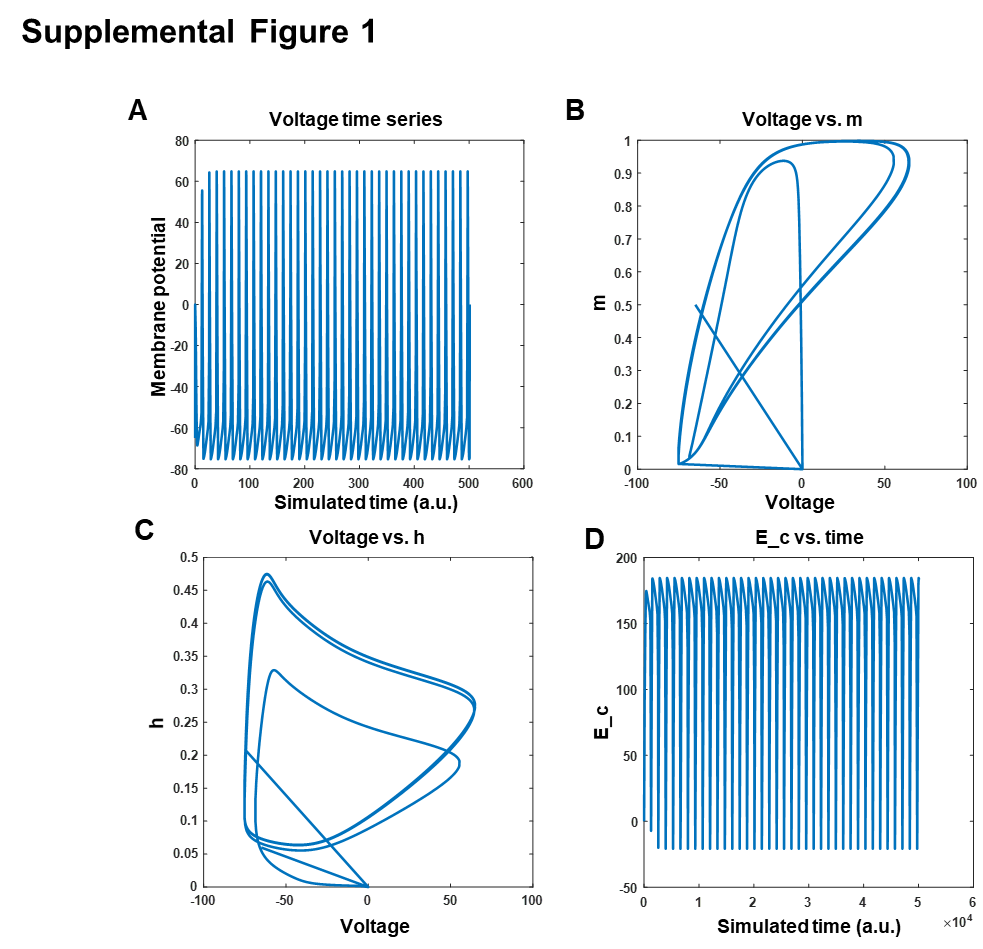
**

**Supplemental Figure 1. Characterization of the energy consumption of a place cell in the implemented Hodgkin-Huxley (H-H) model. (A)** Dynamics of the membrane potential of a simulated neuron. **(B-C)** Dynamics of the membrane voltage when m and h control the Na+ channels indicating the probability that a channel is open at a given moment in time. **(D)** Dynamics of the energy consumed across the simulated time. The energy consumption by a place cell during an action potential was ~188 nJ. All panels were made using custom code in Matlab R2016b (https://www.mathworks.com/). This work is licensed under a Creative Commons Attribution 4.0 International License (CC BY 4.0) (https://creativecommons.org/licenses/by/4.0/).

**Supplemental Figure 2**


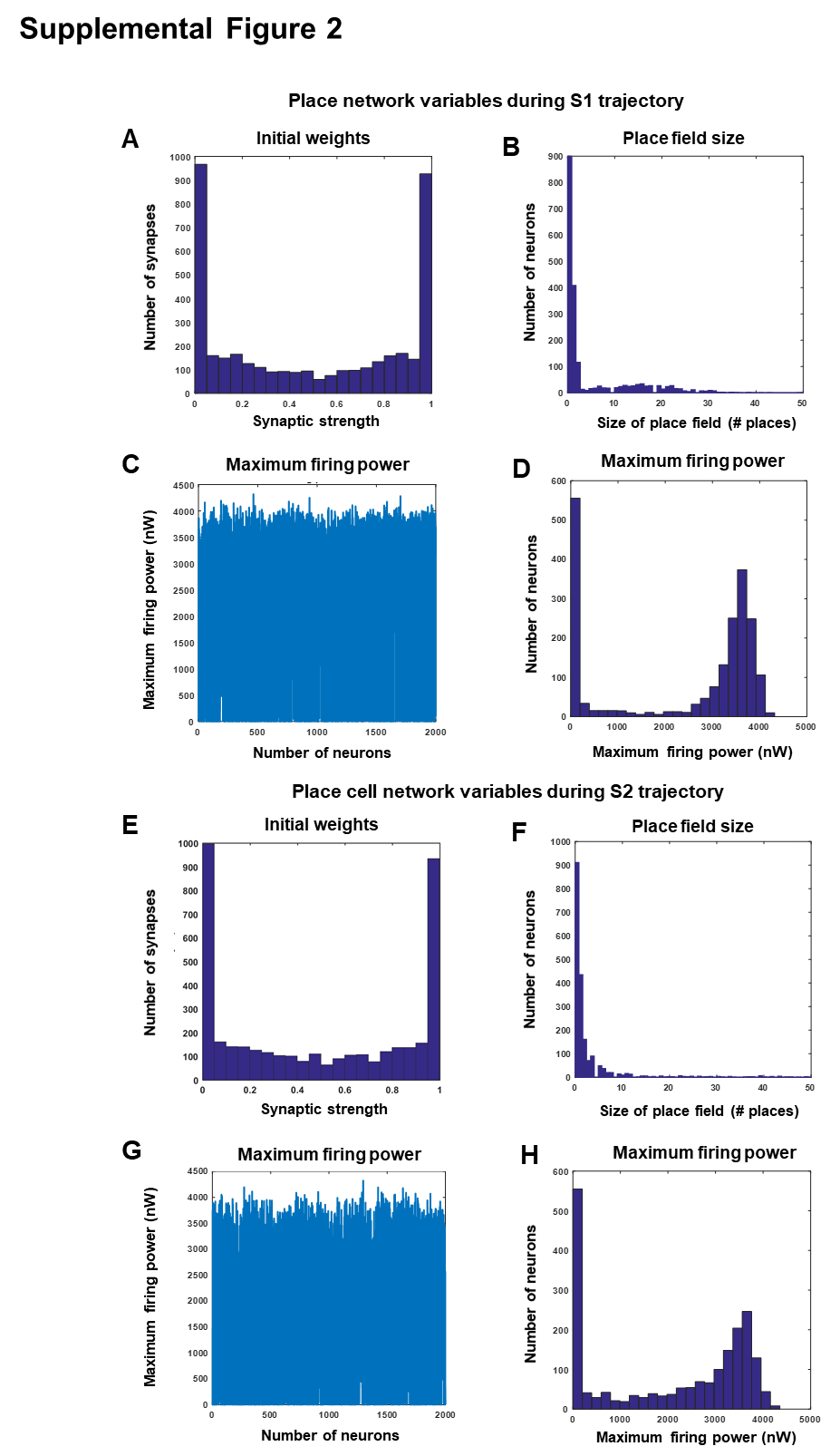


**Supplemental Figure 2. Examples of the distribution of different parameters obtained from the place cell model when considering the S1 and S2 trajectories.** For trajectory S1 after animal’s exploration, **(A)** example of the initial weight distribution. **(B)** Size of place fields after processing all the visual scenes. **(C)** Maximum firing power across place cells. **(D)** Distributions of maximum firing power across place cells. Given the individual differences of place cells (e.g., sensory preferences), the firing power of place cells (C) and the distribution of place fields (D) varied across the network along with the simulation. However, the place field centers were near the maximum firing power of place cells (see Methods). **(E-H)** Similar descriptions for trajectory S2. All panels were made using custom code in Matlab R2016b (https://www.mathworks.com/). This work is licensed under a Creative Commons Attribution 4.0 International License (CC BY 4.0) (https://creativecommons.org/licenses/by/4.0/).

**Supplemental Figure 3**


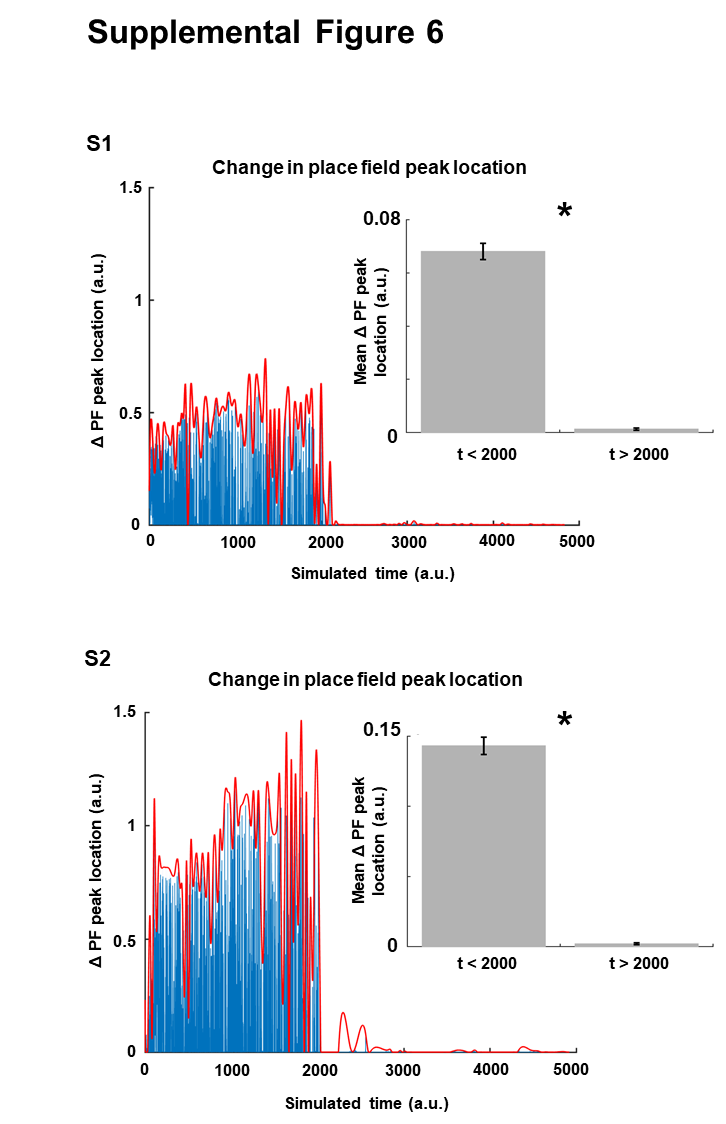


**Supplemental Figure 3. Stability of place field peak locations.** Change in PF peak location (Δ PF peak location) before and after t=2000 for both trajectories. Error bars depicted s.e.m. Differences in the mean Δ PF peak location; S1: p = 9.50e-135, ci = (0.0617; 0.0719), tstat: 25.51, df: 4811, sd: 0.0892; S2: P = 4.74e-153, ci = (0.1310; 0.1513), tstat: 27.3167, df: 4899; unpaired two-sample t-test. ‘ci’ indicates confidence interval, ‘tstat’ represents the value of the test statistic, ‘df’ is the degrees of freedom of the test and ‘sd’ indicates the pooled estimate of the population standard deviation. Blue traces represent Δ PF peak location across time, and red traces indicate the upper peak envelope of the Δ PF peak location considering a 20 a.u. peak separation across time. All panels were made using custom code in Matlab R2016b (https://www.mathworks.com/). This work is licensed under a Creative Commons Attribution 4.0 International License (CC BY 4.0) (https://creativecommons.org/licenses/by/4.0/).

**Supplemental Figure 4**

**
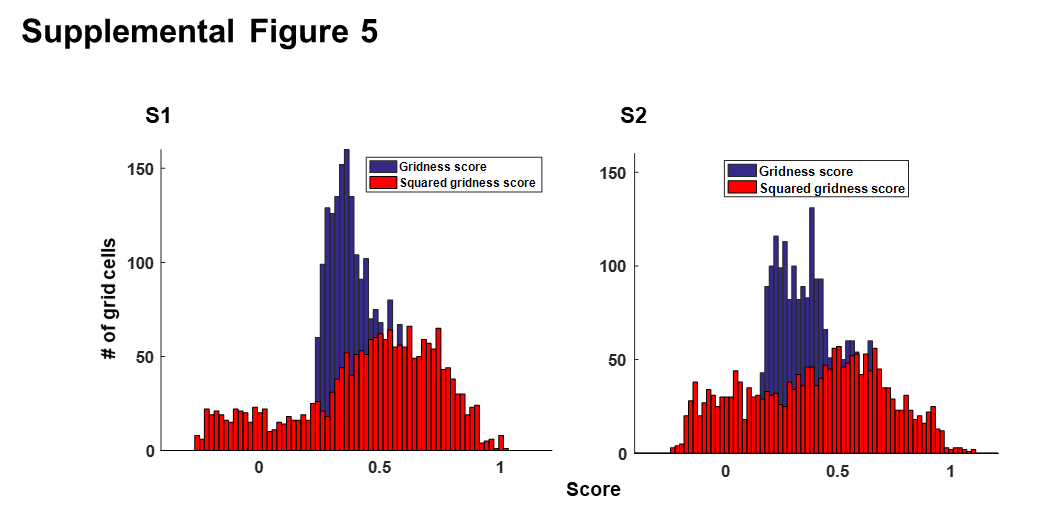
**

**Supplemental Figure 4.** Histograms of gridness values from the grid network are discriminated by trajectory. We used two different measures based on Dordek et al. 2016 ^1^ to compute the gridness score (blue) and the squared gridness score (blue). These results suggested that gridness was present for both trajectories regardless of the used gridness score. All panels were made using custom code in Matlab R2016b (https://www.mathworks.com/). This work is licensed under a Creative Commons Attribution 4.0 International License (CC BY 4.0) (https://creativecommons.org/licenses/by/4.0/).

**Supplemental Figure 5**


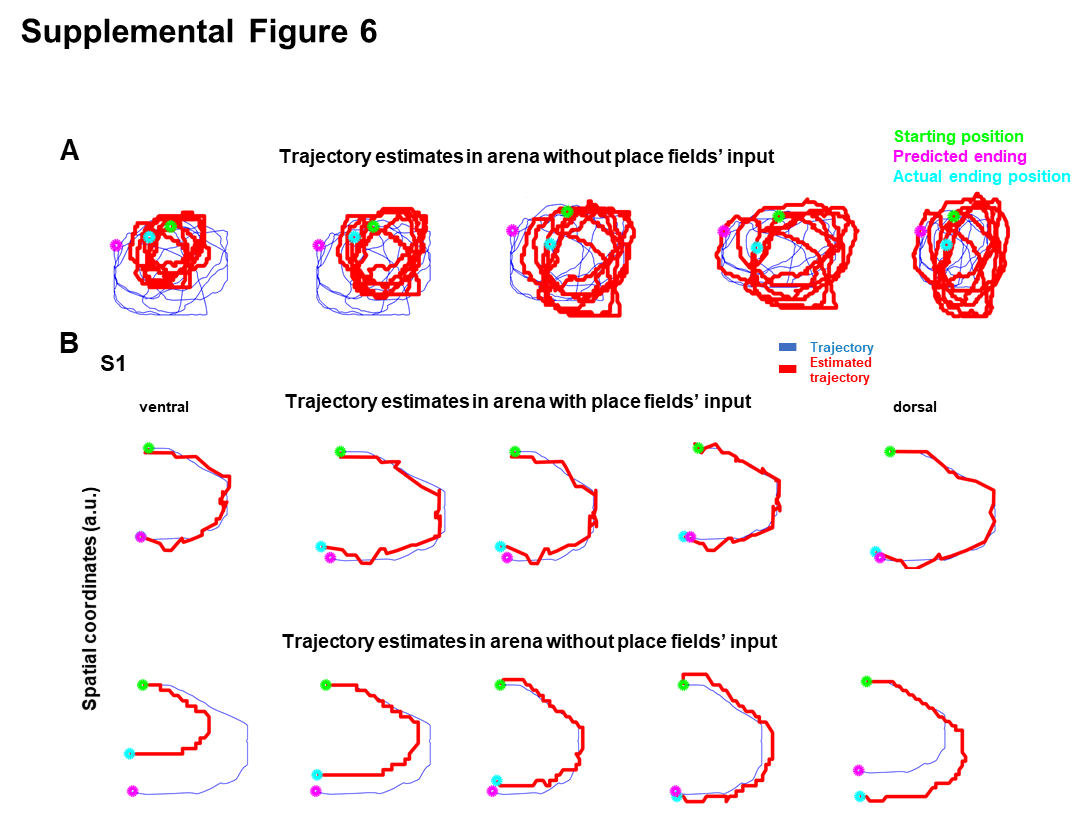


**Supplemental Figure 5. Place field input to grid cells enabled error reduction for path integration. (A)** Representation of the estimated trajectory for each module in the absence of place field inputs for the full trajectory (1000-time steps) shown in **Fig. 4C**. **(B)** Example of the estimated trajectories made by the grid cell modules during a brief S1 movement across the arena for 100-time steps. The top row represents estimations from the starting position in the presence of place field input and the bottom row represents the prediction of each module in the absence of place input. Plots visually suggest a better prediction of the actual animal’s trajectory when place field inputs are provided to grid cells. The green dot represents the starting position, the magenta dot indicates the predicted ending of the trajectory, and the pink dots the actual ending position. All panels were made using custom code in Matlab R2016b (https://www.mathworks.com/). This work is licensed under a Creative Commons Attribution 4.0 International License (CC BY 4.0) (https://creativecommons.org/licenses/by/4.0/).

**Supplemental Figure 6**


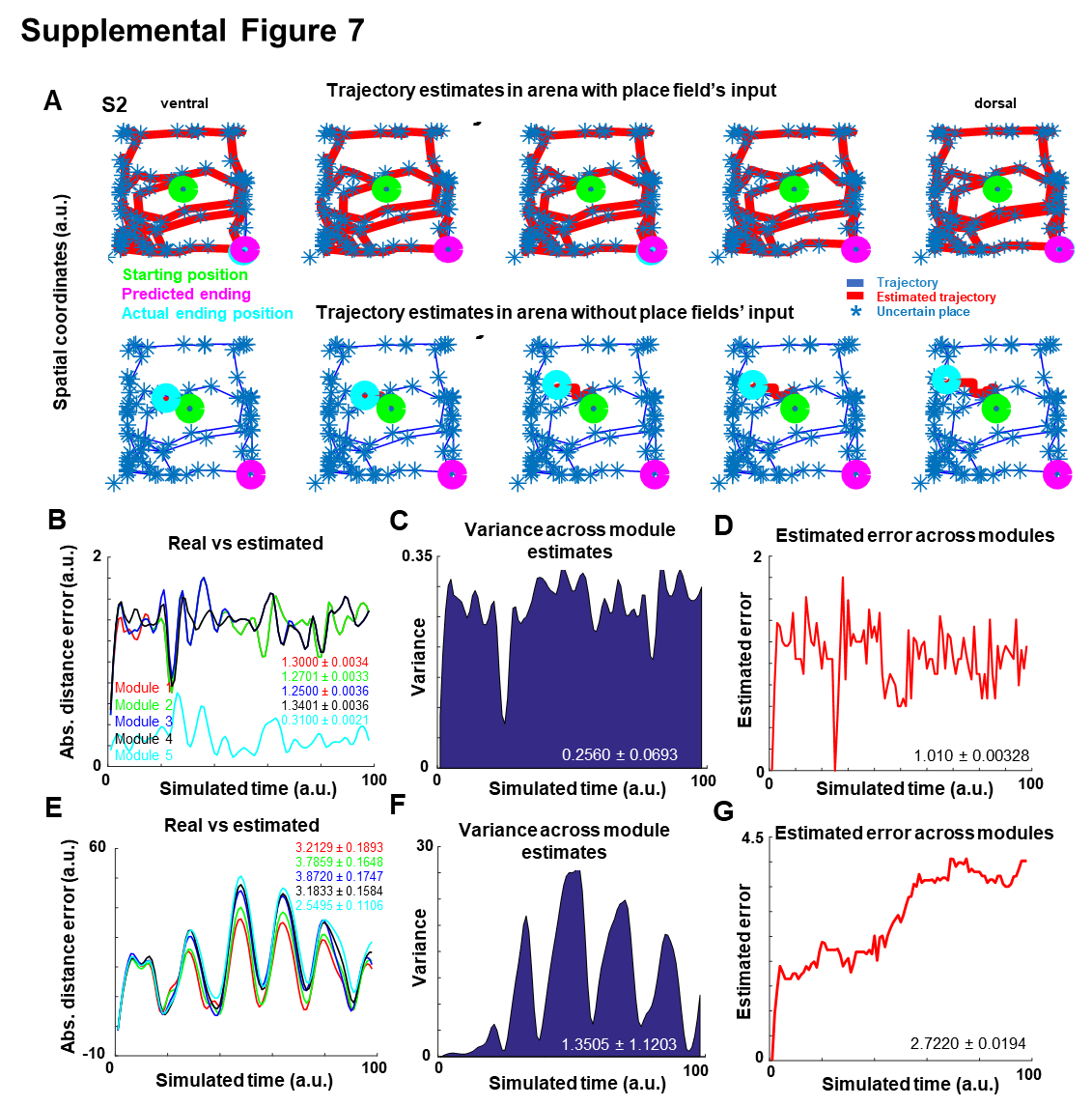


**Supplemental Figure 6. Place field input to grid cells enabled a better error reduction during the S2 trajectory. (A)** Example of the estimated trajectories made by the grid modules during a brief S2 trajectory across the arena. Plots indicated that a better prediction of the actual animal’s trajectory was possible when place field inputs were given to grid cells. **(B&E)** The measure of the absolute distance error based on Euclidean distance comparing the estimated trajectory across modules and the actual trajectory in the presence (B) or absence (E) of place field input. Plots indicated that the distance was higher when place field input to the grid network was absent. **(C&F)** The measure of the variance across estimates showed a similar observation when inputs were provided (C) or absent (F) to the grid network. **(D&G)** Estimated error showing a lower error when (D) place field input was provided and (G) in the absence of input. All panels were made using custom code in Matlab R2016b (https://www.mathworks.com/). This work is licensed under a Creative Commons Attribution 4.0 International License (CC BY 4.0) (https://creativecommons.org/licenses/by/4.0/).**Supplemental Figure 7**

**
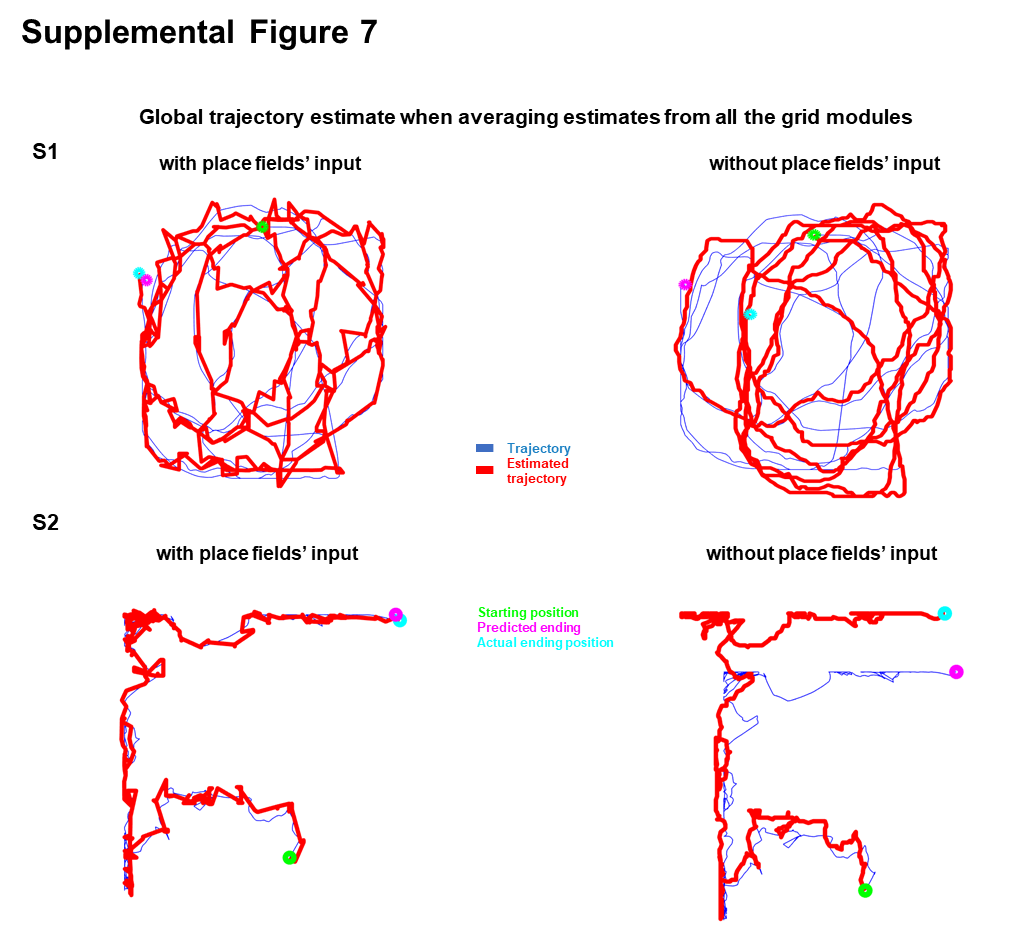
**

**Supplemental Figure 7. Global trajectory estimates presented similar error accumulation without place fields’ input for both trajectories.** Example trajectories when the estimations were computed averaging the estimates from each grid module with or without place fields’ input. Simulated time steps 1000. Plots were divided for S1 and S2 trajectories. The green dot represents the starting position, the magenta dot indicates the predicted ending of the trajectory, and the pink dots the actual ending position. All panels were made using custom code in Matlab R2016b (https://www.mathworks.com/). This work is licensed under a Creative Commons Attribution 4.0 International License (CC BY 4.0) (https://creativecommons.org/licenses/by/4.0/).

**Supplemental Figure 8**


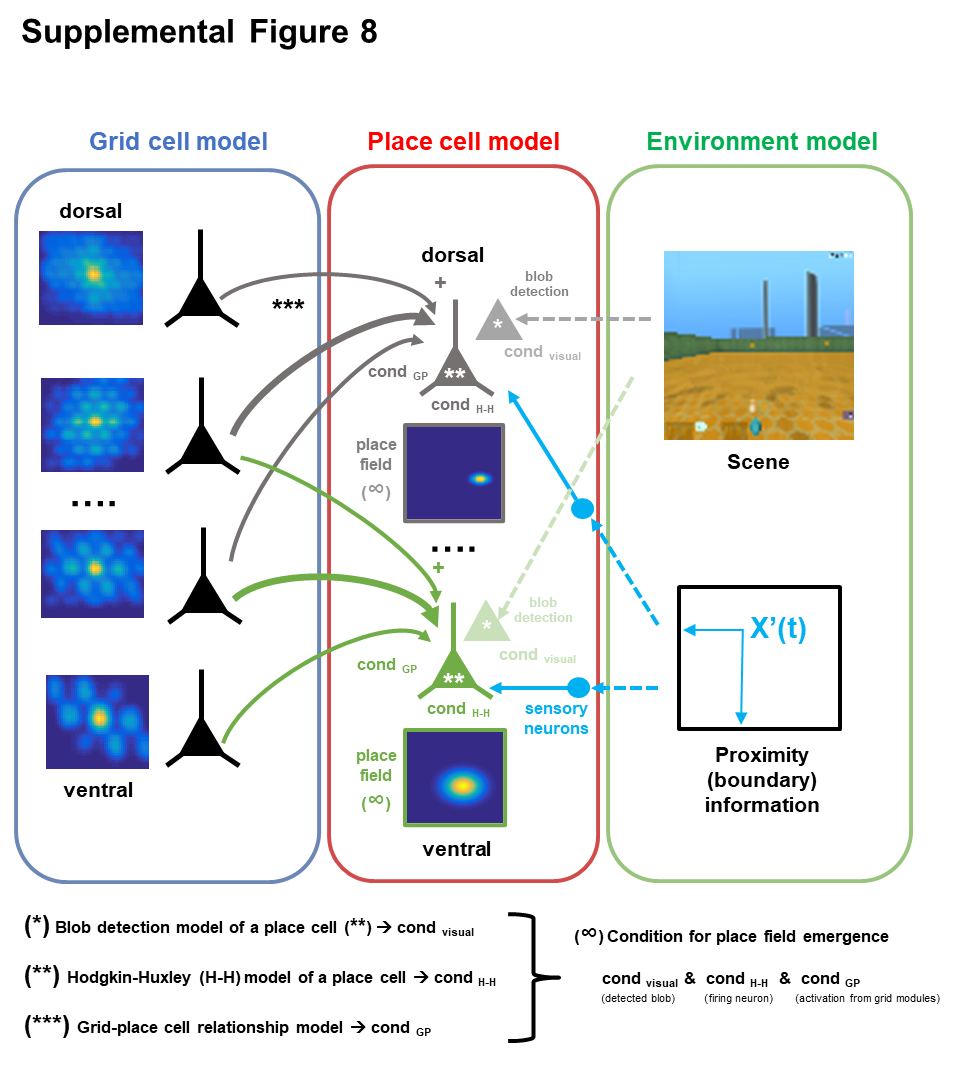


**Supplemental Figure 8. Schematics representing the relationships between the place and grid cell models and the information obtained from the environment model for place field emergence.** Three different conditions must happen simultaneously to place field could emerge. These conditions refer to the detection of a blob (cond _visual_) within the current scene obtained from the environment model, the place cell must fire at that time (cond _H-H_) according to the Hodgkin-Huxley model, and the input activities of grid cell modules (cond _GP_) represented by the connection between the modules and each place cell must go above to certain threshold. This work is licensed under a Creative Commons Attribution 4.0 International License (CC BY 4.0) (https://creativecommons.org/licenses/by/4.0/).

**REFERENCES**

1. Dordek, Y., Soudry, D., Meir, R. & Derdikman, D. Extracting grid cell characteristics from place cell inputs using non-negative principal component analysis. *Elife* (2016). doi:10.7554/eLife.10094
